# What influences birth place preferences, choices and decision-making amongst healthy women with straightforward pregnancies in the UK? A qualitative evidence synthesis using a ‘best fit’ framework approach

**DOI:** 10.1186/s12884-017-1279-7

**Published:** 2017-03-31

**Authors:** Kirstie Coxon, Alison Chisholm, Reem Malouf, Rachel Rowe, Jennifer Hollowell

**Affiliations:** 1 Faculty of Health, Social Care and Education, Kingston University and St. George’s, University of London, 6th Floor, Hunter Wing, St George’s Campus, Cranmer Terrace, Tooting, London, SW17 0RE UK; 2 Policy Research Unit in Maternal Health and Care, National Perinatal Epidemiology Unit, Nuffield Department of Population Health, University of Oxford, Old Road Campus, Oxford, OX3 7LF UK; 3 Currently at Health Experiences Research Group, Nuffield Department of Primary Care Health Sciences, University of Oxford, Radcliffe Observatory Quarter, Woodstock Road, Oxford, OX2 6GG UK

**Keywords:** Place of birth, Systematic review, Decision making, Choices, Women’s views, Women’s experiences, Maternity care, Preferences

## Abstract

**Background:**

English maternity care policy has supported offering women choice of birth setting for over twenty years, but only 13% of women in England currently give birth in settings other than obstetric units (OUs). It is unclear why uptake of non-OU settings for birth remains relatively low. This paper presents a synthesis of qualitative evidence which explores influences on women’s experiences of birth place choice, preference and decision-making from the perspectives of women using maternity services.

**Methods:**

Qualitative evidence synthesis of UK research published January 1992-March 2015, using a ‘best-fit’ framework approach. Searches were run in seven electronic data bases applying a comprehensive search strategy. Thematic framework analysis was used to synthesise extracted data from included studies.

**Results:**

Twenty-four papers drawing on twenty studies met the inclusion criteria. The synthesis identified support for the key framework themes. Women’s experiences of choosing or deciding where to give birth were influenced by whether they received information about available options and about the right to choose, women’s preferences for different services and their attributes, previous birth experiences, views of family, friends and health care professionals and women’s beliefs about risk and safety. The synthesis additionally identified that women’s access to choice of place of birth during the antenatal period varied. Planning to give birth in OU was straightforward, but although women considering birth in a setting other than hospital OU were sometimes well-supported, they also encountered obstacles and described needing to ‘counter the negativity’ surrounding home birth or birth in midwife-led settings.

**Conclusions:**

Over the period covered by the review, it was straightforward for low risk women to opt for hospital birth in the UK. Accessing home birth was more complex and contested. The evidence on freestanding midwifery units (FMUs) is more limited, but suggests that women wanting to opt for an FMU birth experienced similar barriers. The extent to which women experienced similar problems accessing alongside midwifery units (AMUs) is unclear.

Women’s preferences for different birth options, particularly for ‘hospital’ vs non-hospital settings, are shaped by their pre-existing values, beliefs and experience, and not all women are open to all birth settings.

**Electronic supplementary material:**

The online version of this article (doi:10.1186/s12884-017-1279-7) contains supplementary material, which is available to authorized users.

## Background

Choice of place of birth has been part of English maternity care policy since publication of *Changing Childbirth* [1] in 1993, and was reiterated in 2014 in revised NICE guidelines on intrapartum care of healthy women and their babies [2]. Overall, women experience fewer interventions when they plan to give birth in midwifery units or at home [3], appear to have a more positive experience of care [4] and the costs of intrapartum care are also lower [5].

Few other high income nations, with the exception of the Netherlands, actively promote choice of place of birth. Four birth settings are potentially available to women in England: planned home birth, freestanding midwifery units (FMUs), alongside midwifery units (AMUs) and obstetric units (OUs) [2]. The availability of some options, particularly AMUs, has increased in recent years, but the number of FMUs has remained static overall, and recent figures suggest that only 13% of women actually give birth in settings other than hospital OUs [6].

The reasons for low uptake of non-OU birth settings are unclear. Socio-demoraphic characteristics may contribute to this; evidence from the *Birthplace study* [3] suggests that women who planned home birth were more likely than those who planned OU birth to be white, older, speak fluent English and to live in a more affluent area. Other studies suggest that hospital is still perceived to be the normal or ‘default’ option, and that many women consider hospital OU the safest place to give birth [7–10]. There is some evidence that alternatives are not routinely offered, or that the differences between different kinds of setting (such as AMU and FMU) are not fully explained to women [7], although it is not known whether this has changed with the recent expansion of AMU services.

This qualitative evidence synthesis (QES) was conducted to explore UK women’s experiences of choosing or planning where to give birth since the publication of *Changing Childbirth* [1, 11]. It brings together evidence on women’s preferences, choices and decision-making, with a view to identifying the characteristics of UK services and service providers which appear to facilitate choice of birth setting, and any factors which affect women’s decision-making, or their ability to exercise choice. The focus is on healthy women with straightforward pregnancies, in a context where choice is supported by policy in the UK’s publicly funded health system.

## Methods

The synthesis reported here is a component study of the *Birthplace Choices* project, [12] which was designed to inform policy on 'choice' in relation to childbirth. The project included two linked reviews, which used a common protocol and then separated published literature into quantitative and qualitative reviews. This qualitative evidence synthesis (QES) is reported in line with the ENTREQ statement [13], and aimed to address the following research question:

*What influences birth place preferences, choices and decision making amongst healthy women with straightforward pregnancies in the UK?*

### Data synthesis method

**Fig. 1:**
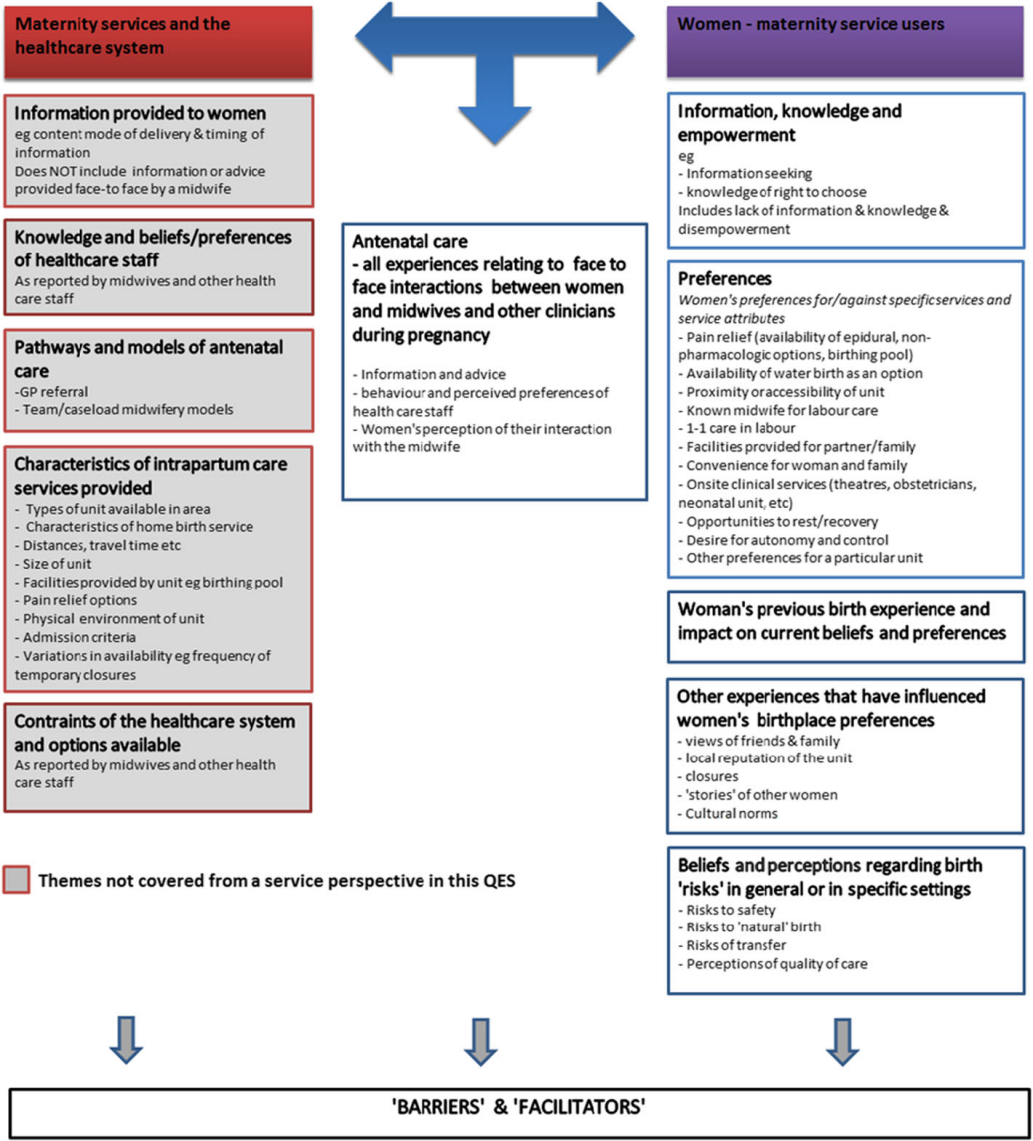
‘Access to care’ model. Model adapted from Khan & Bhardwaj’s (1994) paper [22]: *Access to Health Care. A Conceptual Framework and its relevance to Health Care Planning*. Adapted to birth place decisions and focused to women’s characteristics and perceptions of services for QES framework

Given the policy focus for this QES, we carried out a framework synthesis [14, 15], specifically a ‘best-fit’ framework synthesis [16, 17]. The ‘best-fit’ approach is a novel methodological development, designed to incorporate relevant theories identified in the literature within a framework analysis. The authors of the ‘best fit’ approach suggest the method is ‘suited to producing new conceptual models for describing or explaining the decision-making and health behaviours of patients’ [17] (p.14). The approach requires that researchers identify an a–priori theoretical framework based on published theories or models, against which data from the review is coded. We considered theoretical approaches to decision-making [18–20] and behaviour change [20], but neither seemed directly applicable to the process of birth place decisions. Following Brunton et al’s approach [21], we consulted with policy stakeholders, lay individuals and groups with experience and expertise in the review topic. Following these discussions, we chose to adopt an ‘access’ perspective, drawing on Khan and Bhardwaj’s model [22], on the basis that the overall intention was to inform policy that aims to widen access to choice of place of birth. Interim findings from an initial scoping review were shared with a panel of key stakeholders, user representatives and lay members, and feedback used to make minor refinements to the best-fit model, resulting in a final model (established in July 2015) which formed the basis of the main analysis (see Fig. 1).

### Criteria for study inclusion

We included empirical studies (Jan 1992 to mid-March 2015) which contained qualitative data from qualitative or mixed method design studies on the birth place preferences, choices and decision-making of women users of maternity services. Only UK studies were included because the review was designed to answer a UK policy question, and we felt that there were benefits to understanding women’s experiences in a single well-defined health care system with universal access to a full range of integrated maternity services. As far as possible, we only included data from healthy women with straightforward pregnancies. We included papers which reported on mixed-risk populations, but excluded findings which explicitly related to women with ‘higher risk’ pregnancies (based on NICE criteria [2]). Papers which reported only the views of birth partners or of health care professionals were excluded.

### Search strategy and screening methods

We developed a comprehensive search strategy using a modified SPIDER approach [23] (see Additional file 1). We searched the following databases: ASSIA (Proquest); CINAHL plus EBSCOHost; EMBASE (OvidSP); Medline (OvidSP); PsycINFO (OvidSP); Science Citation Index (Web of Science Core Collection); Social Sciences Citation Index (Web of Science Core Collection). The search strategy aimed to identify studies which explored choice, preference and decision-making amongst healthy women with straightforward pregnancies in relation to place of birth. Papers published in English between January 1992-March 2015 were sought, because these were thought likely to reflect women’s experiences post-*Changing Childbirth*. Systematic reviews and reports which included UK and other countries were used solely to identify additional eligible studies; reference lists of included papers were also searched for additional studies.

### Study selection

Two reviewers independently screened titles, abstracts and full text articles, applying the study eligibility criteria (see Additional file 1 for further details).

### Quality assessment

Two reviewers (AC and KC) appraised included papers using the CASP qualitative checklist [24]. No papers were excluded from the review on the basis of quality.

### Data extraction and synthesis

Two reviewers (AC and KC) extracted descriptive information about the studies using a proforma, cross-checked by RM.

The analytic framework was developed using a-priori themes and sub-themes from the best-fit model, using NVIVO (v10) [25]. Main headings from the ‘Access to care’ model (Fig. 1) were entered into NVivo for the purposes of deductive analysis (e.g. ‘Key theme 1: Information, knowledge and empowerment’). Sub-themes were drawn from the detailed examples beneath the key theme (e.g. ‘information seeking’ was an a-priori sub-theme of Key theme 1). Content from the findings sections of included papers was coded deductively, and data which did not ‘fit’ within the a-priori themes or sub-themes was placed into new inductive codes, and separated wherever possible into the four different birth settings (referring to birth in OU, AMU, FMU or home) for purposes of comparison.

Whilst this QES was in progress, members of the *Birthplace Choices* research team were additionally commissioned to carry out a rapid mixed-methods review of the literature on birthplace choices for the NHS England National Maternity Review [26], findings from which have subsequently been reported [27]. Analysis for this QES was conducted concurrently with the rapid mixed- methods review commissioned by the NHS Maternity Review. Two researchers (KC and AC) separately analysed the same body of qualitative evidence. KC led the data extraction and analysis for this QES and AC conducted the data extraction and analysis for the rapid mixed-methods review. The researchers then conferred, shared findings and discussed observed differences and inconsistencies.

## Results

### Search results

**Fig. 2:**
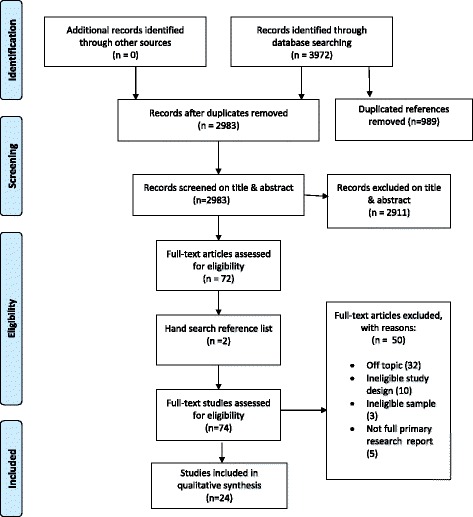
PRISMA Flow Diagram

Out of 2983 records screened, and following checking of references from included studies, 24 full text articles were included in the review (see Fig. 2 PRISMA diagram). Seven papers reported findings from separate aspects of three studies, and in each case the same method was shared across papers. The included papers therefore draw on 20 relevant studies.

### Description of included studies

Included papers are summarised in Table 1, and presented in chronological order. Earlier studies [28, 29] tended to focus on ‘consumer’ choice between either hospital or home birth. Later research used a broader conceptualisation of choice, considering control, birth experience and satisfaction alongside ‘choice’. Most papers focused on home and/or hospital birth, with fewer studies of newer models of care (AMU or FMU). Where authors described settings as birth centres or midwifery units, the authors classified these as AMUs or FMUs based on the descriptive information provided.

**Table 1:** Characteristics of included studies. Studies listed in chronological order

	Aim	Setting	Methods	Analysis	Options available to participants	Sample and sample characteristics
Ogden et al. (1997a and 1997b) [42, 43]	To explore the factors involved in deciding to have a home birth.	London, England	Interviews	Thematic	OU or home	Postnatal (3–5 years) Mixed age and parity
Ogden et al. (1998) [39]	To ‘explore the experiences of women who have a contemporary commonplace hospital birth’.	London, England	In depth interviews	Thematic	OU or home (in principle)	Postnatal (within 5 years) Multiparous women
Mansion and McGuire (1998) [28]	To explore what influences women in their choice of DOMINO birth.	Central Scotland	Interview study	Thematic	Hospital OU or DOMINO	Antenatal *n* = 8 women 22–37 weeks pregnant aged 21–35. Mixed parity.
Tinkler and Quinney (1998) [48]	To explore women’s experiences of being informed and making decisions (including choice of place of birth).	England	Mixed: interviews (*n* = 8) and focus groups (*n* = 14)	Content and thematic	Choice of OU or home birth.	Antenatal and postnatal *n* = 68 Diverse socio-economic status (SES), age, parity
Emslie et al. (1999) [29]	To examine the way women make choices and decisions about choice of place of birth	North -East Scotland	Interviews (longitudinal)	Grounded theory Thematic analysis	OU, AMU, FMU and home birth (although home birth discouraged)	Antenatal and postnatal follow up. *n* = 20 mixed parity.
Longworth et al. (2001) [35]	To identify 'valued attributes’ of home and hospital birth for women	England (London)	Focus groups (*n* = 2)	Analysis not described	Home or OU	Antenatal or postnatal – booked in preceding 12 months *n* = 20 women participated No sample characteristics provided.
Cheung (2002) [40]	Identify experiences of Chinese and Scottish childbearing women in Scotland	Scotland	Interviews (longitudinal)	Comparative thematic analysis	Hospital OU or home	Antenatal and postnatal follow- up *n* = 20 women (Chinese *n* = 10, Scottish *n* = 10) Age 25–82. Mixed parity.
Stapleton et al. (2002) [37]	To examine the use of evidence-based leaflets on informed choice in maternity services.	Wales (13 maternity units)	Observations (*n* = 886), field notes, interviews.	Grounded Theory thematic analysis	Home birth and OU	Antenatal and postnatal Antenatal interviews (*n* = 85) Postnatal interviews (*n* = 78) *NB Figures relate to entire study, not limited to Choice of Place of Birth leaflet*. Theoretical sampling including diverse age, ethnicity and obstetric experiences but parity not specified.
Madi and Crow (2003) [38]	To find out how much information women have about hospital and home birth	S England	Interviews	Grounded theory thematic analysis	Home or OU	Antenatal. *n* = 33 (20 planning home birth, 13 planning hospital birth) 32–42 weeks pregnant Low risk pregnancies. Mixed parity.
Watts et al. (2003) [47]	An evaluation of how the new midwife led service (FMU) was meeting women’s needs, from the service user perspective.	England (rural)	Unstructured interviews, observations and documentary research	Thematic analysis	Home, FMU or OU	Postnatal (within 1 year) *n* = 8 Multiparous, low risk women
Andrews (2004) [33]	Explore women’s experience of home birth	S Wales	Interviews	Phenomenological thematic analysis	Hospital OU or home	Postnatal (up to 6 m) *n* = 8 Mixed parity
Lavender and Chapple (2005) [41]	Identify models of care that meet the needs of women and offer choice of place of birth.	England	Survey, 71% response rate. Only responses to open questions within survey included.	Thematic analysis	Hospital OU, AMU, FMU, Home (National survey, options varied depending on area where participant lived).	Antenatal (mean 29 weeks) *n* = 2071 women who used one of 12 maternity units in England Mixed parity mean age 29, mostly white/English speaking, diverse SES
Shaw and Kitzinger (2005) [32]	To document the obstacles women encounter in trying to exercise their right to choose to give birth at home.	UK	Documentary data. Call transcripts and emails to the Home Birth helpline.	Content and thematic analysis	Hospital OU – home birth was an option but callers had experienced barriers to this.	*n* = 56 callers to Home Birth helpline, of whom *n* = 54 women calling on their own behalf. No other demographic data was gathered.
Barber et al. (2006) [34]	Identify factors that influence women’s decisions about where to give birth	S England	Focus groups (*n* = 5)	Content and thematic analysis	Hospital OU, FMU or home	Pregnant women (29–40 weeks). *n* = 20 women (Mixed parity)
Walsh (2006) [46]	Ethnographic exploration of culture, beliefs, values, customs and practices in FMU.	England (Midlands)	Participant observation (*n* = 15, 6–10 h),interviews with women (*n* = 30, 5 of whom were observed)	Thematic analysis	FMU or OU, home.	Antenatal and postnatal (around 3 m) *n* = 40 Mixed parity.
Jomeen (2007) [31]	To explore and advance the understanding of maternity care choice through women’s experiences.	North England	Longitudinal narrative interviews	Thematic analysis	OU, home birth, FMU (new).	Antenatal and postnatal follow up. *n* = 10 low risk women Parity not specified. Aged 18 or older.
Houghton et al. (2008) [8]	To explore the rationale behind women’s choice of place of birth and the influences on their decision-making.	NW England	Observations and semi-structured interviews	Thematic analysis	OU, home birth, AMU (*'integrated MLU*')	Antenatal and postnatal follow up *n* = 30 Mixed parity aged 18–39, 80% married/cohabiting, diverse SES
Pitchforth et al. (2008) [36]	To explore women’s perceptions of different models of care and willingness to make ‘trade-offs’ in remote and rural areas.	Scotland Remote & rural	Focus groups (*n* = 8, range 4–7 participants)	Thematic analysis	OU, remote FMU or home	Postnatal women (1 m – 7 years) *n* = 47 participants Aged 24–45 Parous.
Pitchforth et al. (2009) [9]	To explore women’s perceptions of “choice”	Scotland Remote & rural	Focus groups (*n* = 12, range 4–9 participants)	Thematic analysis	OU, remote FMU or home	Postnatal (within 4 years) *n* = 70 Mixed parity.
McCutcheon and Brown (2012) [44]	To add to the body of knowledge on place of birth and home birth experiences.	England	Interview study	Thematic analysis	Hospital OU or home	Postnatal Women (*n* = 9) aged 27–78 who had given birth at home at least once (*n* = 7) or only in hospital (*n* = 2). Diverse ethnicity.
Newburn (2012) [45]	To examine lived experiences in a new birth centre (AMU)	England	Ethnography (participant observation)	Thematic analysis	AMU home or OU	Antenatal (observation) and postnatal (interviews) Postnatal women (*n* = 11) Mixed SES, ethnically diverse. Parity not clear, but includes multiparous women.
Coxon et al. (2014) [10]	To understand better what accounts for birth place preferences.	S England	Interviews (longitudinal)	Narrative analysis (thematic and structural)	Hospital OU and home (all); AMU and/or FMU depending on local services.	Antenatal with end of pregnancy follow up. *n* = 41 women Aged 19 to 42. Mixed parity, mixed ethnicity, diverse SES, mixed risk
Coxon et al. (2015) [30]	To explore the influence of pregnancy and birth experiences on planned place of birth in future pregnancies	S England	Interviews (longitudinal)	Narrative analysis (thematic and structural)	Hospital OU and home (all); AMU and/or FMU depending on local services.	Antenatal with postnatal follow up *n* = 41 women Aged 19 to 42. Mixed parity, mixed ethnicity, diverse SES, mixed risk

Most studies reported recruiting relatively small, purposive samples, and usually included participants of mixed parity. Eighteen out of twenty four papers addressed OU birth, nineteen addressed home birth and seven addressed birth in FMUs (three of which referred to FMUs in remote or rural areas). Whilst six papers referred to AMUs as a setting available to participants, only four of these papers presented qualitative evidence about women’s experiences of choosing AMUs. Twelve papers included data from women who could choose between OU, home and a midwifery unit. Later papers were more likely to have used longitudinal or mixed methodologies. Some included explicit reference to feminist or social science theoretical perspectives [10, 30–32].

### Study quality

The CASP appraisal (see Additional file 2: Table S1) found that five papers had quality issues in reporting. Reflexivity and explanation of data analysis were the items most often lacking sufficient detail [28, 33–36].

### Findings

**Fig. 3:**
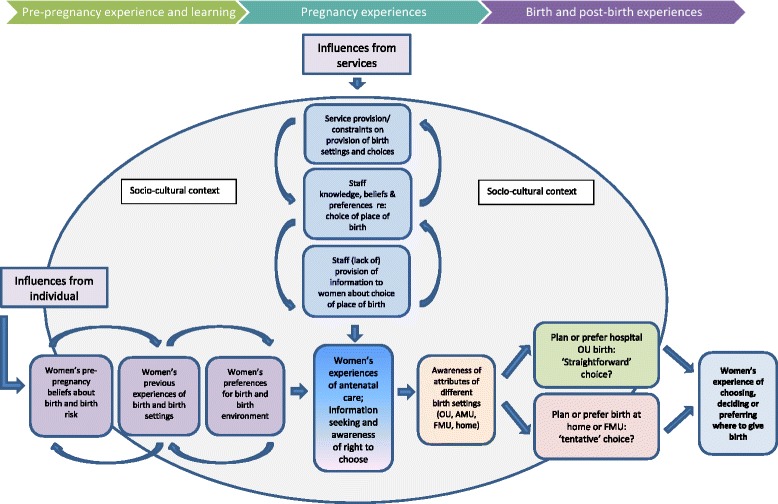
UK women’s experiences of choosing, preferring or deciding where to give birth (New Conceptual Model)

This synthesis was designed to explore women’s experiences of planning, choosing or deciding where to give birth. We start by briefly summarising findings from five of the a-priori themes arising from our original ‘best fit’ model (see Fig. 1). Findings relating to some of these themes have already been reported as part of the rapid mixed-methods review conducted for the NHS England National Maternity Review [27] and these are therefore not the main focus, but are presented here as context for the new findings. We then go on to present the novel information arising from this synthesis, which arises from new inductive sub-themes within the sixth a-priori theme of women’s antenatal experiences. These revealed some differences in antenatal care experiences for those women who were inclined towards opting to plan birth in an OU, compared with those who might consider planning birth in a non-OU setting. These data have been incorporated into a new conceptual model, which is a development of the original ‘best fit’ model (Fig. 3).

A-priori themes: *How Information, knowledge and empowerment, preferences, previous birth experiences, other experiences and beliefs about risk affect women’s experiences of planning or deciding where to give birth.*

Here, we summarise findings from the deductive analysis using a-priori themes; more detailed information, including quotes from included papers, is provided in Table A, Themes 1–5 (Additional file 3). The findings showed that women often did not feel they had a choice of place of birth, or believed their choice was limited to deciding between two or more hospitals [8, 9, 31, 34, 37, 38]. Women described needing to actively seek out information, especially if they were considering birth in a non-hospital setting [31, 37, 38] (see Table A, Additional file 3, sub-themes 1.1 and 1.2).

A further finding was that many women felt that hospital was the safest setting for birth, and that this was the normal or expected birth setting [8, 10, 39, 40] (Table A, Additional file 3, sub-themes 2.1 (a), 4.3, 5.1). Key attributes which contributed to the sense of safety were access to medical staff and facilities, pain relief being available and not needing to transfer [8, 10, 36–38, 41, 42]. Women’s accounts often drew on their own previous experiences of birth [8, 32, 33, 43], and some felt that if something were to go wrong, giving birth in hospital would protect the home from being a site of bad memories [8]. Nevertheless, hospital OUs were also perceived to be anxiety-provoking or impersonal [28, 35, 44] and, for women in remote and rural areas, the distance and travel time to OU were off-putting [9] (Table A, Additional file 3, sub-theme 2.1 (b)).

In most papers, home birth was the alterative setting available, although some papers did include FMU or AMU birth. Key attributes which make birth in non-OU settings attractive are that the setting is more relaxed, women expect to feel more in control, to manage better, to have family around them and a carer they know [10, 28, 34, 35, 42, 44–47] (Table A, Additional file 3, sub-themes 2.2 (a), 2.3 (a), 2.4 (a)). Some women discussed concerns about transfer from FMU to OU [29, 31]. Women who planned home birth focused on the homely environment and support, and argued that access to transfer enhanced the safety of home birth [8, 43].

#### New sub-themes: *Antenatal experiences and finding out about choice of place of birth*

Two new inductive sub-themes, ‘Finding out about choice of place of birth’ and ‘Making the decision’, were created within the ‘Antenatal care’ a-priori theme during the best-fit framework analysis. Together these captured the depth and extent of evidence in the papers about women’s antenatal experiences. Drawing on data within these themes, it became clear that planning or preferring a particular place of birth is often not a simple ‘one off’ decision made at a specific time point such as the ‘booking’ appointment, as clinical guidance tends to imply [2]. Women’s inclination or preference for the kind of birth they want and aspects of the birth environment seem to be informed by pre-pregnancy beliefs, experiences and upbringing. For some women this inclination may be open to change, while for others it may result in a clear preference for a particular setting prior to birth. At present, there is little evidence about whether women’s preferences are fixed, or alter in response to events during pregnancy. The new conceptual model (see Fig. 3) begins to incorporate these uncertainties and to reflect that preferences or expectations for birth develop over time. The influence of the socio-cultural context of birth is also recognised in the model. We present data to support these changes here.

#### Making sense of differing expectations

To date, research has tended to examine which birth setting choices are available to women, and why these might appeal, but often fails to explore the extent to which women are open to planning birth in different settings. This was an important issue within the synthesis, because through comparing evidence from women who were inclined towards birth in a hospital setting with data from those more open to non-OU birth, we identified evidence that women who prefer or plan birth in OU settings experience this as a straightforward or ‘taken for granted’ choice, without an explicit decision-making process. In Madi and Crow’s research [38] (p.333), women had a choice of hospital or home birth but still opted to give birth in hospital as a ‘default’

*We just assumed it would be in hospital, we didn’t really talk about it. We didn’t discuss it at all.*

Those who were open to considering or preferred non-OU settings (usually home birth in the included papers) described having a more difficult time, as their preference often meant making a case or using persuasion. Amongst women with a preference for or inclination towards home birth the situation was more complex, as another of Madi and Crow’s participants explained [38]:

*I think a lot of them* (referring to healthcare professionals (HCPs)) *make an assumption and think well, yes, you come under (name of hospital/OU)…and say, ‘you will be going along there to have the baby, won’t you?’ And, people, unless they have specifically thought about it and are willing to state, ‘well, actually, no, I won’t,’ then they will not get the option at all.*

These accounts reflect evidence in the included papers that there was a difference between these two perspectives (that is, preference for or a pre-existing inclination towards either OU or home birth). Understanding these preferences is likely to be important when considering how to improve and promote access to choice of place of birth. For this reason, we present findings from these differing perspectives in the sections below.

### The experiences of women who were open to considering, or preferred, birth at home, or in a non-hospital setting

#### ‘Countering the negativity’

The predominant experience reported by women planning birth in a setting other than hospital was one of finding their preference challenged on several fronts, and therefore we discuss this experience first, before considering the experience of women who did receive support for their preference and outlining what factors or features of care were helpful to them.

In interview data (See Table B, Additional data file 3 for themes and sample quotes), women described how home birth was positioned as counter-cultural, hippy, non-normative or alternative [8, 40, 43, 44] or that home was not the right setting for birth [8, 41]. Within this context, women described that clinicians, friends and family might oppose planned home birth [10, 32], explained that they needed positive support from their partners to achieve home birth [33, 38, 43] and that they felt ultimately responsible for this decision and any consequences [9, 37].

However, the response from HCPs, as well as from family and friends, was often cautious, and women described having to ‘counter the negativity’ [33] (p.520) surrounding home birth [32, 33, 37, 43], having to be strong or brave to pursue their preference, or feeling ‘embattled’ [9, 30, 32, 33, 44] (see Table B, Additional file 3, sub-theme 6.2 (a)). This perception was related to HCP responses to their requests for information or advice about home birth [8, 31, 32, 41, 43, 44] or FMU birth in some cases [8, 34]. Midwives were described as providing little information or not offering home birth [8, 9, 31, 32, 37, 38, 44] or AMU birth [29, 31, 45] or ‘blocking’ discussions by use of body language or conversation closure [8, 9, 28, 31, 32, 37, 38, 40, 43, 44, 48]. Some women reported being told that they were not allowed to have a home birth [9, 34] or that HCPs (including GPs and midwives) described them as selfish or reckless, or asking for a costly NHS service [31, 32, 37, 49]. Even when in-principle approval for non-OU birth was granted, women described having to repeatedly demonstrate suitability for a midwife-led birth [8, 31, 33, 44], giving rise to a sense that the decision was always tentative and subject to review. Women were also told that the service might not be available due to lack of staff [44] (see Table A, sub-theme 4.4 and Table B, sub-theme 6.3 (a), both in Additional file 3).

In view of these experiences, we propose that the socio-cultural context (See Fig. 3) is important because it informs both women’s perceptions of birth settings, and the responses women receive from partners and HCPs. Women who prefer or would consider home birth found they had beliefs and value systems which were sometimes at odds with those of HCPs, families and friends who had a ‘risk averse’, or ‘better safe than sorry’ philosophy (see Table B, Additional file 3, sub-theme 6.1 (a)). However, the data above has been presented to demonstrate a comparison, which runs the risk of over-stating the resistance women might encounter to home or non-OU birth. Many women did receive at least some level of support for preferences consistent with planning birth in non-OU settings, and we discuss this below, drawing out the factors which seem to enhance women’s access to choice of place of birth.

#### Receiving positive support for birth at home or in non-OU settings

Women who received more positive support towards planning or preferring birth at home or in non-OU settings reported different experiences. In these papers, women described preferring home or non-OU birth for the reasons already outlined (see Table A, Additional file 3, sub-themes 2.2 (a), 2.3 (a) and 2.4 (a)). Although they were bound to experience the same ‘risk averse’ sociocultural context as women who encountered resistance, certain factors made their preferences seem more achievable. One positive influence was knowing somebody else who had given birth at home [8, 38, 43] or in an FMU [46]. Husbands, partners, family or friends could then also be supportive of the home birth plan [38, 43], perhaps because this was more ‘normal’ in their locality or family. Some had their own previous positive experiences of home [30, 33, 42, 43] or FMU birth [46] to draw upon, and this was an important resource. Women found it empowering to successfully give birth at home [44], and for some who had previously attempted FMU birth and been transferred to hospital, FMU was still a preferred setting in the current pregnancy [41] (see Table B, Additional file 3, sub-themes 6.1 (b) and 6.2 (b)).

A key difference was that women felt supported in their preference by their HCP; their midwife or GP gave them information and actively offered them a home birth [8, 33, 38] or supported them in their choice of home birth [8, 33]. Women trusted their midwives [44, 45, 47], and felt confident that their midwife had the right skills if needed, including emergency care [8, 41, 43]. They also valued having a known midwife or doula for birth at home [10, 28, 35, 44] or FMU [9, 29, 36, 41] (see Table B, Additional file 3, sub-theme 6.3 (b)). These factors were associated both with a willingness to consider non-OU birth in the first place, and with a sense that this would be acceptable to family and HCPs.

Encountering negativity or a more positive support for home birth might occur together or at different time-points in pregnancy. In practice, most women experience elements of each, and some described having very supportive families and midwives. Nevertheless, the predominant reported experience was one of resistance to non-OU birth settings, particularly home birth (see Table B, Additional file 3, sub-themes 6.1 (a), 6.2 (a), 6.3 (a)), and this impeded access to choice of place of birth.

### The experience of women who plan or prefer OU birth

Wherever women planned to give birth, the prevalent sociocultural narrative or expectation that OU is taken for granted as the ‘right place’ to give birth was influential [8, 10, 30, 32, 33, 35, 47], and this view generally aligned well with the beliefs and values of HCPs (see Table A, Additional file 3, sub-themes 5.1 and 5.2). Perhaps for this reason, when women expressed preference for hospital birth, there was very little evidence that HCPs sought to establish whether they were aware they had a choice, and none to suggest that women’s preference for hospital birth was at any time challenged. Women did not describe having their choice to birth in hospital re-examined as pregnancy progressed, even though the data all relates to women at low risk of complications, who might be well placed to consider birth in non-OU settings.

The cultural presumption towards hospital was facilitative for women who instinctively preferred OU birth; it provided a dependable and widely accepted explanation that OU is the safest and best equipped setting for birth, being clean, clinical, with full access to pain relief and medical, midwifery and specialist staff, and escalation to more acute care without the need for transfer [8, 29, 31, 35, 40, 41]. The same view also led to non-OU settings being easily discounted by women who preferred OU as potentially unsafe, lacking essential staff or equipment or inconvenient [8, 41].

## Discussion

The QES found that few women considered that they were given a ‘real’ choice of place of birth. Planning birth in hospital was considered straightforward and uncontested during pregnancy, such that it was often not considered a ‘choice’ at all, whereas a decision to give birth at home was more often experienced as tentative and uncertain throughout pregnancy and even during labour and birth. There was some evidence that this is also the case for planned FMU birth, but although six included papers came from settings where AMUs were an option, these contained little discussion of women’s experiences of choosing AMU or accessing information about AMU services. For this reason, it is unclear whether planned AMU birth provokes the same uncertainties.

Non-OU services were often not routinely discussed at booking [8, 9, 31, 32, 37, 38, 44]. Although non-OU birth was sometimes a supported choice [8, 9, 38, 43] women described needing to proactively request information from HCPs.

This synthesis also provided confirmatory evidence that past birth experiences, beliefs about birth risks and safety, preferences for particular service attributes (such as ‘medical facilities’ or ‘relaxed, surroundings’) and the views of family, friends, partners and health care professionals influence women’s experience of choosing where to give birth. We have presented a new conceptual model to incorporate the new observations made in our QES into the original ‘best fit’ model. We acknowledge that this is an incremental development, that further confirmation (and refutation) is needed, and that the model proposed is likely to need to be revised as gaps in the evidence are addressed by new research. A particular area which requires more clarity is the extent to which women’s preferences for birth and the birth environment are ‘set’, or might be something that women willingly revisit if they are presented with clear information about the choices open to them, and about the various attributes and potentials of different settings for birth. New evidence about women’s experiences of choosing where to give birth in localities where AMUs are provided would also be valuable to inform the model.

This QES was a component study undertaken as part of the *Birthplace Choices* project, conducted in parallel with a rapid mixed-methods review for the National Maternity Review [27]. The rapid mixed-methods review [27] provides additional detailed evidence – qualitative and quantitative- relating to women’s preferences for specific service attributes. For example, the quantitative evidence showed that many women had a clear preference for birth in a hospital setting where medical staff were readily available although not necessarily directly involved in their care [50]. Distance and travel time were also important, with women preferring local services. Other factors identified as important to women in the quantitative systematic review, such as access to pain relief options, continuity of care and transfer issues, were also found in this QES.

We have argued here that choosing or planning to give birth in non-hospital settings was often experienced by women as a tentative or uncertain choice. In their recent systematic review of evidence on midwives’ place of birth discussions with women, Henshall et al. [51] found that midwives felt under pressure to recommend hospital OU birth for organisational reasons. The same review found that midwives provided varying levels of information depending on their confidence in discussing non-OU birth options and their personal beliefs about the appropriateness and feasibility of alternative birth settings. These findings have resonance with women’s perceptions in our QES that health care professionals were not always positive about non-OU options, and did not routinely provide full information about locally available services.

### International evidence

Our findings relate directly to UK maternity care, but similar issues have been identified in other countries. Much of the evidence reviewed here relates to choosing between home or FMU and hospital, in a context where these choices are supported, and so these findings may have relevance to other countries where efforts are being made to provide women with choice of birth at home or in FMUs, including the Netherlands, Denmark, Australia, New Zealand and Canada.

In their international review of women’s perception of birth choice, Hadjigeourgiou et al. [52] also identified that concerns about obstetric safety were prioritised, and that women struggled to assert their autonomy when they wished to plan birth at home. A prospective mixed-methods New Zealand study found similar perspectives on risk and safety in relation to different birth settings to those identified in this QES, but additionally reported that women in New Zealand consistently receive continuity of midwifery care and felt they were the ‘principal birthplace decision-maker’ [53]. The same team also published research proposing that women who chose to give birth in an FMU felt confident in their birthing ability, in their midwife and in the FMU service [54]. Recent research on primary birth units (similar to FMUs) in Canada [55] and Australia [56] also details the persistence of an obstetric risk-based approach in low risk maternity populations, and identifies trusting relationships between women, HCPs and service provider organisations as a basis for safe, supportive care.

#### Strengths and limitations of the review

This ‘best fit’ framework QES used a structured search strategy with clear inclusion and exclusion criteria (Additional file 1) and the thematic analysis was based on a theoretical framework drawn from the literature and refined in response to expert opinion from a stakeholder panel including representatives of service user groups and lay members.

The QES was conducted from the perspective of women, so does not consider the beliefs and experiences of HCPs, which may be important to understand. Grey literature and other unpublished reports were not included, primarily to ensure that the review findings represent relevant, peer-reviewed evidence which is reported in a way that makes quality appraisal feasible. However, additional searches of doctoral theses or similar would have identified additional evidence in some of the areas where the evidence we found was sparse. For example, our Medline search did not identify peer-reviewed NIHR reports, and one important report was subsequently identified [57]. This report contains useful data on women’s reasons for choosing AMU, and on responses to ‘opt-in’ pathways, where women have to book for an AMU birth, and ‘opt-out’, where AMU is the default option for eligible women. We discuss the findings of this study further below.

The inclusion of studies published since 1992 can be considered both a strength and a limitation. The consistency of certain findings over time added confirmatory value; for example, there was evidence that women experienced resistance to non-OU birth in both early and later papers. On the other hand, as the CASP appraisals identified, there were limitations in the methodological quality of some included papers. Earlier papers were subject to less stringent standards of reporting than would currently be acceptable, often did not report details of data analysis or reflexivity and had varying levels of engagement with conceptual theory or interpretative epistemology, reflecting historical changes in the conduct and reporting of qualitative research over time.

Much of the research in this review was undertaken before the publication of the *Birthplace cohort study* findings on the safety of midwifery-led settings and the subsequent update of the NICE guideline [2, 3]. Some of the negativity encountered by women may have reflected the then-current lack of evidence on this topic, and clinicians’ concerns about safety, and it is possible that women are now encountering more support for birth in non-hospital settings. Recent multi-site focus group research undertaken by members of the *Birthplace Choices* team has explored women’s experiences of choice of different settings since the update of the NICE guideline, which now recommends that women at low risk of complications ‘may choose any birth setting (home, freestanding midwifery unit, alongside midwifery unit or obstetric unit)’ and should be advised that midwifery-led settings are ‘particularly suitable’ for them [2]. This research may help determine whether the difficulties women identified in this QES persist.

Most of the evidence in this QES comes from studies involving women choosing between an OU and home birth. This makes it difficult to disentangle whether women prefer a hospital birth, have an aversion to or fear of home birth, or whether OU birth is simply the ‘default option’. The included studies were mostly undertaken before the recent rapid expansion of AMU provision in England [6, 58], and only four included papers contained qualitative data about choosing AMUs. This scarcity of data meant we were unable to draw conclusions about women’s experiences of choosing or preferring AMU, and it remains unclear whether women consider AMUs to be part of ‘hospital OUs’ or whether they are seen as being more similar to FMU or home settings; future research might usefully address this gap in the evidence.

In their report on an organisational study of four English AMUs, McCourt et al. [57] identified that women were aware of differences between AMU and OU facilities, although they sometimes received inconsistent information from midwives. This report also details the different approaches used by services to encourage uptake and normalisation of AMUs, including, for example, policies whereby healthy women with straightforward pregnancies are automatically referred to AMUs unless they ‘opt out’. McCourt et al. [57] present some evidence about this from women’s perspectives, and suggest that in the areas they studied, women were more likely to receive information where an ‘opt out’ model was in place. There is little evidence about whether women who wish to have an OU birth experience barriers in areas where the AMU is the default (‘opt out’) option for low risk women.

#### Implications for policy and practice

This review suggests that women do not receive consistent, balanced information about the birth settings options which are available to them, and whilst some of the papers may reflect past or dated practice, reports that access to birth in non-OU settings were not always well supported by HCPs were also present in the more recent papers. There is now good evidence of safety and policy support in the UK for choice of place of birth for healthy women with straightforward pregnancies, and a more structured and consistent approach to information provision by HCPs (particularly midwives, GPs and obstetricians) is likely to enhance women’s access to choice of place of birth.

Women valued receiving information from midwives and from other HCPs, and wanted to feel confident that their HCP would support them in their choices. This suggests that HCPs need to work in a context which is supportive of their providing choice of place of birth and facilitates sufficient time to engage with women’s views and perspectives, to routinely share information about the different options available and to respond to women’s questions.

Women were concerned if they heard that a home birth service might not be provided, due to lack of staff or to resource issues. The provision of a range of options in localities coupled with support for service sustainability in the long term will help women to feel confident that the service will be available to them. As familiarity appears to reduce resistance to non-traditional settings, this might also help such services to be considered normal or usual for eligible women who plan home birth, or birth in other non-OU settings.

The timing of information provision is also important. As birth place preferences may change over the course of pregnancy, choice might be better supported by ensuring that women have the opportunity to discuss their options at different points during pregnancy. It is also important that midwives are able to explain the risks and benefits of different settings for birth to help women decide which setting might best meet their individual needs and preferences.

Finally, in the context of recommendations policymakers and commissioners of services may want to consider the findings of this review and the other linked reviews [27, 50] when reconfiguring maternity services. In particular, findings that women generally have a preference for local services but may not all be open to non-hospital settings may present a challenge as obstetric services are centralised, since this could potentially leave women in some areas having to choose between local ‘non-hospital’ services (home or FMU) and more distant hospital-based services (OU or AMU).

## Conclusion

Despite a national policy of offering women choice about place of birth, the evidence from studies conducted between the mid-1990s and 2010 shows that it was straightforward for low risk women to opt for hospital birth in the UK. Accessing home birth was more complex and contested and although the evidence on FMUs is more limited, it suggests that women wanting to opt for an FMU birth experienced similar barriers. Most of the evidence predates the recent expansion of AMU provision and the extent to which women experienced similar problems accessing AMUs is unclear.

Our findings suggest that women’s preferences for different birth options, particularly for ‘hospital’ vs non-hospital settings, are shaped by their pre-existing values, beliefs and experience, and that not all women are open to all birth settings.

## Additional files

Additional file 1: Search strategy and structure and additional detail about selection. (DOCX 32 kb)

Additional file 2: Table S1. CASP appraisals. Two reviewers (KC and AC) appraised included papers using the CASP qualitative checklist (http://www.casp-uk.net/). The first reviewer (KC) conducted a full CASP appraisal, and a second reviewer (AC) independently conducted a modified CASP appraisal focusing on the adequacy of reporting, following Carroll et al.’s method [1]. The reviewers resolved areas of disagreement following initial reviews; these were minor and reflected variation in degree to which a paper met a given criteria, rather than conflicting views about the paper. No papers were excluded from the review on the basis of quality. (DOCX 41 kb)

Additional file 3: Examples of data and evidence to support a-priori themes (Tables A and B). (DOCX 50 kb)

## Abbreviations

AMU: Alongside Midwifery Unit
CASP: Critical Appraisal Skills Programme
FMU: Freestanding Midwifery Unit
HCP: Health Care Professional
OU: Obstetric Unit
QES: Qualitative Evidence Synthesis
