# Supplementary material for: What influences birth place preferences, choices and decision-making amongst healthy women with straightforward pregnancies in the UK? A qualitative evidence synthesis using a ‘best fit’ framework approach
Source: BMC Pregnancy Childbirth. 2017 Mar 31;17:103. doi: 10.1186/s12884-017-1279-7 (PMC5374625; doi:10.1186/s12884-017-1279-7)
Supplement: Supplementary file 1 — Search strategy and structure and additional detail about selection. (DOCX 32 kb) [file 12884_2017_1279_MOESM1_ESM.docx]

**Additional file 1: Search and study selection strategy**

The qualitative systematic review of birth place preferences was one component of a broader systematic review which also encompasses the quantitative evidence relating women’s birth place preferences. Some aspects of the methods reflect the fact that searches were common to the two reviews.

**Scoping review**

An initial scoping review was undertaken to map available evidence, to clarify the methodology and scope of the evidence synthesis, to test eligibility criteria and to pilot search terms for the review. The scoping review confirmed that there was relevant, published qualitative evidence on this topic, sufficient to undertake a synthesis of qualitative evidence.

The scoping review also revealed a high volume of survey and other quantitative research, some of which appeared relevant to the review question, but which often lacked detail about the reasons underpinning women’s decision-making about place of birth. The scoping review findings were discussed with the co-investigator group, and a decision was taken to systematically identify all relevant primary research, in order to map the full scope of available evidence. The qualitative evidence synthesis then focused on qualitative research, which was thought most likely to answer the question posed, through providing detailed and contextual data on women’s experiences of choice of place of birth. The team also conducted a second separate systematic review of quantitative/mixed methods evidence. A benefit of searching and screening the qualitative and quantitative studies together was that this was both more efficient and likely to improve the ascertainment of mixed methods studies.

**Inclusion/exclusion criteria for QES**

**Study design**

1. Qualitative primary research studies which explore choice, preference and decision-making in relation to place of birth.
2. As described above, the team also systematically identified and retrieved quantitative studies on this topic. Data from these studies were not included in the qualitative synthesis, but were used to inform interpretation of the synthesis, and to reflect on other published evidence in the discussion of findings.

Systematic reviews, qualitative syntheses and other reports including studies conducted in multiple countries including but not limited to the UK were systematically identified during screening but used solely to identify additional eligible studies.

**Date and source**

1. Published in a scientific journal 1992-mid March 2015.

**Study Participants**

1. Studies in which participants are ‘low risk’ women who have used UK maternity services during pregnancy (‘eligible women’).

Studies with an unselected participant group, or where there is evidence of both low and high risk cases within the participant group, will only be included if it appears feasible to separately identify data from eligible women within the study findings.

**Phenomenon of interest**

1. Studies which were designed to generate knowledge about any aspect of women’s beliefs, preferences, knowledge and experience affecting their choice of place of birth during pregnancy, including issues relating to the availability of services, and characteristics of maternity services and staff.

Topics relevant to this review include, but are not limited to:

- Women’s experiences of choosing a planned place of birth during pregnancy (for example, information provided; how and whether choices were presented; how and whether women were facilitated or supported to make a choice).
- Women’s experiences of decision-making regarding planned place of birth.
- Women’s reasons for choosing or preferring one maternity unit over another or of choosing one birth setting over another (e.g. home birth vs. OU).
- Information on what criteria women used to differentiate between different birth settings.
- Women’s preferences for a particular attribute of a unit or service.
- Barriers to exercising choice that that women have experienced.
- Any services provided that have helped women access their preferred choice, including access to private maternity services.
- Women’s perceptions of timing of planned place of birth decisions or choices, or of opportunities to revise or change their planned place of birth.
- Women’s perceptions of different birth settings (for example, furnishings, equipment, staffing, ease of access, perceived barriers to access)
- Sources of information upon which women draw.
- Women’s perceptions of risk in relation to different birth settings.

The search strategy was designed to identify research studies within the scope of the broader review irrespective of study design and screening was conducted by sequentially applying the criteria applicable to each component of the review as described below. Researchers who were part of the broader research team included Kirstie Coxon (KC) who with Jennifer Hollowell (JH) led the qualitative review, Alison Chisholm (AC), a qualitative researcher, and Joanna Forsey (JF), a midwife and MRes student, were involved in screening, in addition to KC, JH and Reem Malouf (RM).

**Search strategy for QES**

Search structured using SPIDER search strategy [1]

| **SPIDER tool** | **Search Terms relating to:** |
| --- | --- |
| Sample | Women who have used maternity services |
| Phenomenon of Interest | Place of birth setting (OU, AMU, FMU, home) |
| Design | Any [initial search conducted for mixed methods syntheses] |
| Evaluation | Choice, option, preference, experience, perspective, decision |
| Research setting | UK NHS or private maternity care |

| **SPIDER Tool** | **Search Terms relating to:** |
| --- | --- |
| **S**ample | **Pregnant women**  Matern* or pregnan* OR women Pregnant women/ Mothers/ |
| **P**henomenon of **i**nterest | **Maternity unit/midwifey unit/birth centre/home birth/intrapartum careplace of birth**  Maternity adj2 (care or unit* or setting? Or center? Or centre? Or hospital? Or service*) obstetric adj2 (unit? or center? Or centre?) midwi* adj2 (unit? or center? Or centre?) Home birth* or home childbirth or home delivery birth adj2 (unit? or center? Or centre? Or place) intrapartum care  Place of birth/ Birthing Centers/ Delivery Rooms/ Home Childbirth/ |
| **D**esign | NA |
| **E**valuation | **Preferences/choice/experiences/decisions/views/influences/experiences/ attitudes/expectations**  Prefer* or choice* or choos* or option? Or decision* or decid* or view* or experience* or need* or suggest* or influenc* or attitude* or satisf* or value* or expectation* or inform* or advice*or consum* or “Consumer –led” |
| **R**esearch setting | **United Kingdom/Great Britain/England/Scotland/Northern Ireland/United Kingdom/British/NHS**  United kingdom or uk or britain or gb or england or wales or scotland or northern ireland or british or nhs or national health service or Great Britain |

***Databases searched***

- Applied Social Science Index and Abstracts (ASSIA)[Proquest]
- Cumulative Index to Nursing and Allied Health (CINAHL) plus [EBSCOHost]
- EMBASE [OvidSP]
- Medline [OvidSP]
- PsycINFO [OvidSP]
- Science Citation Index [Web of Science Core Collection]
- Social Sciences Citation Index [Web of Science Core Collection]

Databases were searched from 1992 to mid-March 2015.

**2. Screening and study selection**

Two reviewers independently screened titles and abstracts and full text as required. As noted above, because this review was conducted as one component of a broader systematic review the screening was conducted by sequentially applying the criteria applicable to each component of the review, with reviewers working in pairs. Screening was conducted as follows:

**Stage 1:** Jennifer Hollowell (JH) and Joanna Forsey (JF) screened all titles and abstracts applying only the exclusion criteria relating to country, broad topic of research, type of report and study population. Only references independently excluded by both reviewers were excluded. Remaining references were rescreened in stage 2.

**Stage 2:** Remaining tiles/abstracts (n= 487) were re-screened by Kirstie Coxon *(*AA) and Jennifer Hollowell (JH) who applied the screening criteria for the broader review, but did not exclude studies solely on the basis of design (i.e. qualitative mixed-methods and quantitative studies were included). Discrepancies were resolved by discussion. Full-text articles (n=72) were retrieved for the remaining studies.

Stage 3 (qualitative review); JH and KC read and independently screened the full-text article to identify eligible qualitative and mixed methods studies. Discrepancies were resolved by discussion, with a third reviewer, Alison Chisholm (AC), involved as required. During this screening process, JH and KC flagged all potentially eligible quantitative and mixed-methods studies.

Stage 4 (quantitative review): JH and Reem Malouf (RM) independently screened the full-text articles flagged at stage 3 to identify eligible quantitative studies.

**References**

[1] Cooke A, Smith D, Booth A: **Beyond PICO: the SPIDER tool for qualitative evidence synthesis**. *Qualitative health research* 2012, **22**(10):1435-1443.
