# Supplementary material for: What influences birth place preferences, choices and decision-making amongst healthy women with straightforward pregnancies in the UK? A qualitative evidence synthesis using a ‘best fit’ framework approach
Source: BMC Pregnancy Childbirth. 2017 Mar 31;17:103. doi: 10.1186/s12884-017-1279-7 (PMC5374625; doi:10.1186/s12884-017-1279-7)
Supplement: Supplementary file 2 — CASP appraisals. Two reviewers (KC and AC) appraised included papers using the CASP qualitative checklist (http://www.casp-uk.net/). The first reviewer (KC) conducted a full CASP appraisal, and a second reviewer (AC) independently conducted a modified CASP appraisal focusing on the adequacy of reporting, following Carroll et al.’s method [1]. The reviewers resolved areas of disagreement following initial reviews; these were minor and reflected variation in degree to which a paper met a given criteria, rather than conflicting views about the paper. No papers were excluded from the review on the basis of quality. (DOCX 41 kb) [file 12884_2017_1279_MOESM2_ESM.docx]

**Additional file 2: Full CASP appraisals**

Two reviewers (KC and AC) appraised included papers using the CASP qualitative checklist (<http://www.casp-uk.net/>). The first reviewer (KC) conducted a full CASP appraisal, and a second reviewer (AC) independently conducted a modified CASP appraisal focusing on the adequacy of reporting, following Carroll et al.’s method [1]. The reviewers resolved areas of disagreement following initial reviews; these were minor and reflected variation in degree to which a paper met a given criteria, rather than conflicting views about the paper. No papers were excluded from the review on the basis of quality.

|  | **Q1 Was there a clear statement of the aims of the research?** | **Q2 Was a qualitative methodology appropriate?** | **Q3 Was the research design appropriate to address the aims of the research?** | **Q4 Was the recruitment strategy appropriate to the aims of the research?** | **Q5: Were the data collected in a way that addressed the research issue?** | **Q6: Has the relationship between researcher and participants been adequately considered?** | **Q7: Have ethical issues been taken into consideration?** | **Q8: Was the data analysis sufficiently rigorous?** | **Q9: Is there a clear statement of findings?** | **Q10: How valuable is the research?**  **(1. Contribution to literature and 2. Transferability)** |
| --- | --- | --- | --- | --- | --- | --- | --- | --- | --- | --- |
| Andrews A. Home birth experience 1: decision and expectation. British Journal of Midwifery 2004; 12 (8):518-23. | Yes: explore women's experience of home birth | Yes | Yes: qualitative interviews to explore experiences | Yes: women approached by midwives. Selection rationale unclear (no inclusion/exclusion criteria) but sample appears purposive and appropriate. | Yes: semi-structured interviews in women's homes. Topic guide provided. Taped and transcribed. | No: the author is identified as the interviewer but reflexivity is not discussed. | Yes; ethical approval gained. | Unknown.  Framework of analysis based on phenomenology provided.  No info about selection of themes or reflexivity. Insufficient data provided. | Yes; findings are explicit. Insufficient evidence for subthemes. | Yes (1) & to some extent (2); transferability to other areas planning increase in home birth |
| Barber T, Rogers J, Marsh S. The birth place choice project: phase one. British Journal of Midwifery 2006;14 (10):609-13. | Yes 3 aims including 'to identify factors that influence women's decisions about where to give birth' | Yes | Yes: Focus groups with women (5 groups, 20 women) and midwives (2) conducted to supplement survey data. No further justification of method given. | Yes - pregnant women 30/40 or more invited to participate in FGs via invitation packs given out at clinics. | Unknown - little information about FG process or questions but we know that the FG discussions generated data about 'thoughts and feelings' around information on choice and decision making | No - paper does not specify who facilitated focus groups. | Unknown (not in paper) | Unknown - content analysis by 2 project managers. Data on 3 categories of information identified (similar to framework approach, analysis based on existing survey findings) Insufficient information in paper. | Unknown - qualitative findings used to supplement quant survey so findings arise from Quant survey | 1. Valuable - first UK study to look at women's perspectives of different birth settings 2. Valuable to some extent - quality limitations but balanced by available choices (all 4 choice options available) |
| Cheung NF. Choice and control as experienced by Chinese and Scottish childbearing women in Scotland. Midwifery 2002;18(3):200-13. | Yes - Identify experiences of Chinese and Scottish childbearing women in Scotland (with focus on choice and control) | Yes | Yes | Yes: purposive, and snowballing | Yes; four sequential in-depth interviews with each participant. | Yes | Yes; ethical approval gained and consent from hospital managers to conduct study. | Unknown - some of the analysis is explained; taped, transcribed, filed but method of analysis not explicit | Yes - 'medicalised childbirth' includes section on women's views about place of birth | Yes 1 & 2 but should reflect this is a comparison of Scottish and Chinese women |
| Coxon K, Sandall J, Fulop NJ. To what extent are women free to choose where to give birth? How discourses of risk, blame and responsibility influence birth place decisions. Health, Risk and Society 2014; 16 (1):51-67. | Yes - to understand better what accounts for birth place preferences | Yes | Yes: longitidinal narrative method | Yes: purposive sampling of women with a range of birth place options. | Yes: in depth semi-structured narrative interviews | Unknown: not discussed within paper. | Yes: REC and RD approval | Yes; narrative thematic analysis described. | Yes | Yes 1 and 2 Consider diverse sample and care context |
| Coxon K, Sandall J, Fulop NJ. How do pregnancy and birth experiences influence planned place of birth in future pregnancies? Findings from a longitudinal, narrative study. Birth: Issues in Perinatal Care Feb 2015  42(2), 141–8 | Yes - explored the influence of pregnancy and birth experiences on women’s intended place of birth in current and future pregnancies | Yes | Yes; follow up interviews during a longitudinal prospective study. | Yes: purposive sampling | Yes: three in depth narrative interviews with each participant | Unknown: not discussed within paper. | Yes; ethical approval and RG approval gained | Yes; thematic narrative analysis and structural narrative analysis. | Yes | Yes 1 and 2 Consider diverse sample and care context |
| Madi BC and Crow R. A qualitative study of information about available options for childbirth venue and pregnant women's preference for a place of delivery. Midwifery 2003; 19 (4):328-36. | Yes - to find out how much information women have about the availability of home and hospital as childbirth venues, and how their midwives are involved in helping them make their choice of where to give birth. | Yes | Yes | Yes - low risk women (n=33) planning hospital or home birth recruited via community or hospital midwives. Theoretical sampling used. | Yes; unstructured interviews in women's own homes | Unknown: not discussed within paper. | Yes: ethical approval and RG approval gained. | Yes: GT study, open coding, constant comparison. A researcher not involved also did analysis: agreed with findings. | Yes | Yes 1 and 2; valuable that sample specified low risk women |
| Emslie MJ, Campbell MK, Walker KA, et al. Developing consumer-led maternity services: A survey of women's views in a local healthcare setting. Health Expectations 1999;2(3):195-207. | Yes - To examine the way women make choices and decisions about maternity care and the factors which influence decision making, including choice of place of birth, choice of lead professional and choices in labour management. | Yes | Yes. Not clearly justified however. | Yes. Interview recruitment invitations via survey questionnaire; stratified by parity and geographical area. Eligibility for study (survey & interview) unclear but appears to be low risk women in catchment area | Unknown - no topic guide or similar | no - 'study researcher' description is the only information provided | Unknown - no information in paper. | Unknown - too little information in paper re qualitative analysis. | No; qualitative data is used to provide reasons for quantitative survey findings; no evidence of thematic analysis or synthesis. | Valuable to literature (1) as early study of 4 choice options. Appears transferable but caution in weighting due to lack of information about qualitative study. |
| Watts, K., Fraser DM, and Munir F. The impact of the establishment of a midwife managed unit on women in a rural setting in England. Midwifery 2003;19 (2):106-12. | Yes: An evaluation of how the new midwife led service (FMU) was meeting women's needs, from the service user perspective. | Yes | Yes: case study approach using mixed methods. | Unknown - convenience sample drawn from survey respondents but authors note some women who were not eligible were invited to participate by a third party. Not clear whether this includes interview sample. | Yes - unstructured interviews in women's own homes. Few details of approach; no schedule (unstructured). | No; no indication who did interviews. | Yes: research ethical approval gained for both sites. | Unknown: details of thematic analysis are provided but no themes are presented. | No; qualitative data is used to provide reasons for quantitative survey findings; no evidence of synthesis of qualitative findings. | Unknown (1). Adds to literature from remote and rural areas but sampling, design and analysis limitations are present. 2. Transferability may be limited to remote and rural areas. |
| Houghton G, Bedwell C, Forsey M, et al. Factors influencing choice in birth place -- an exploration of the views of women, their partners and professionals. Evidence Based Midwifery 2008;6(2):59-64. | Yes: study to explore the rationale behind women’s choices and the influences on their decision making. | Yes: authors wished to *explore the rationale behind women’s choices and the influences on their decision making(p.3)* | Yes Qualitative interpretative approach was adopted. Not further justified | Yes Purposive sample, stratified by parity and IMD | Yes - participant observation of first (booking?) appt. Questionnaires x 3 (before booking at 34 weeks and PN). Interviews 34 weeks and PN | No Booking observations were by a research midwife. Not clear who conducted interviews with women | Yes: hospital Trust and local research ethics committee approvals. Anonymity and confidentiality addressed | Unknown - thematic analysis of interviews; constant comparison used. Insufficient information in paper. | Yes but caution because at times women’s and professional’s views are talked about together and not always much data to support conclusions | Yes (1) and (2) with limitations - little known about sample, and conclusions drawn from both professional and women's data - makes sense in context of the research design however |
| Jomeen J. Choice in childbirth: a realistic expectation? British Journal of Midwifery 2007;15 (8):485-90. | Yes: to explore and advance the understanding of maternity care choice through women's experiences. | Yes | Yes - longitudinal in depth narrative interviews | Yes, low risk purposive sample (eligible for midwife led care) recruited, however, insufficient details provided. Women recruited were a subset from a larger cohort study. | Yes - in depth narrative interviews in hospital or at home. Tape recorded. | Yes - reflexivity discussed. | Yes - ethical and RG approval gained. | Unknown. Inductive thematic analysis but scant detail, and no discussion of rigour. | No. The findings are arranged around the theme of 'choice' but no discussion of different themes or synthesis. | 1. Valuable contribution to literature; demonstrates complexity of choice. 2. Transferability - valuable as women had choice of hospital, home or midwife led unit. |
| Lavender T, Chapple J. How women choose where to give birth. Practising Midwife 2005;8 (7):10-5. | Yes: Identify models of care that meet the needs of women and offer choice of place of birth. | Yes - but only used to a minimal extent (open questions within a survey) | Yes - survey plus open questions | Yes; purposive, designed to include women with a broad range of choices. | Yes, open questions within a survey study. | No - not interview data, survey responses only | Yes approvals from research ethics committees, R&D managers and audit coordinators. | Unclear; Thematic analysis of open responses including two researchers to increase reliability of findings but no qualitative theoretical basis. | No; qualitative evidence is used to explain survey findings; no evidence of qualitative synthesis. | Yes (1). Directly relevant data Yes (2) although unclear what choices the women whose quotes were used have. |
| Longworth L, Ratcliffe J, Boulton M. Investigating women's preferences for intrapartum care: home versus hospital births. Health & Social Care in the Community 2001;9(6):404-13. | Yes: To identify 'valued attributes' of home and hospital birth for women, as a precursor to a conjoint analysis study which aimed to identify the relative importance of different attributes to women. | Yes | Yes, to some extent the focus group design was appropriate, although in depth interviews may have been more useful to identify what was important to individals. | Unknown - no details provided | Yes - two focus groups with 10 women; one group comprised women 'booked' to give birth in hospital, and the other women who had booked home birth, in the previous 12 months. May have been AN or PN; not clear. | No: no indication of who conducted focus groups. | Unknown - the subsequent survey study was reviewed by an ethics committee but it is not clear whether this application included the development work. | No | Unknown; four statements from each group are provided, but no quotations or discussion of how these were arrived at. | Unknown value to literature (1) due to lack of information about method. Findings to appear to resonate with other research so likely to be transferable. |
| Mansion EM, McGuire MM. Professional issues. Factors which influence women in their choice of DOMINO care. British Journal of Midwifery 1998; 6(10):664-68. | Yes: To explore what influences women in their choice of DOMINO birth, and identify why there was low uptake of this option. | yes | yes - exploratory study | Yes; convenience sample of women who had chosen DOMINO | Yes - in depth interviews, topics discussed in paper | No - not discussed. | Yes - ethical approval obtained | Unknown; discussion of conceptual analysis and categories but little detail provided | No clear statement of how findings identified or how they relate to each other. | Unknown  1. Valuable as little is written about DOMINO care 2. Limited value as Domino care is not widely offered at present |
| McCutcheon R, Brown D. A qualitative exploration of women's experiences and reflections upon giving birth at home. Evidence Based Midwifery 2012;10(1):23-28. | Yes - to add to the body of knowledge on place of birth and home birth experiences | Yes | Yes: grounded theory interviews | Yes; theoretical sampling of women who had experiences of home birth and two 'disconfirming' cases (women who had given birth in hospital only) | Yes: in depth semi-structured interviews | No - not discussed | Yes; university ethical approval gained (non-NHS recruitment). | Unknown; technique of conceptualising categories using constant comparative method. Not clear whether any measures were used to ensure rigour. | yes | Yes (1) and (2) - but note that with the exception of two women, the sample was selected on basis of having had home birth. |
| Newburn M. The best of both worlds - Parents' motivations for using an alongside birth centre from an ethnographic study. Midwifery 2012;28 (1):61-66 doi: 10.1016/j.midw.2010.10.014. | Yes - 'To examine 'lived experiences in a new birth centre from the perspectives of... parents' | Yes | Yes (observations and interviews) | Yes; purposive | Yes; observation, participant observation, field notes, interviews with women and partners. | Yes | Yes; university and NHS trust ethical approvals and R&D approval gained. | Unknown; clear account of analysis but no information about rigour checks. | Yes; these are organised around a theme of 'deciding where to give birth'. Limited evidence of data synthesis. | Yes, valuable to (1) and (2); few papers on AMU experience exist. |
| Ogden J, Shaw A, Zander L. Women's experience of having a hospital birth. British Journal of Midwifery 1998;6(5):339-45. | Yes; 'to explore the experiences of women who have a contemporary commonplace hospital birth' | Yes | Yes; in depth interviews with women who had given birth in hospital | Yes, purposive: GPs asked to identify women who fitted inclusion criteria (had given birth in hospital 3-5 years previously, not CS). | Yes, in depth interviews using interview guide, in women's homes, by experienced research nurse, tape recorded and transcribed. | No, no discussion other than a description of the researcher ('experience research nurse') | No; ethical review is not mentioned. | Unknown - some discussion of thematic analysis; themes and categories listed but limited information about the analysis, or rigour checks. | No - no clear statement of how findings identified or how they relate to each other. | Yes - valuable to (1) because few papers specifically explore preference for hospital. Valuable to (2) as findings resonate with contemporary research on same topic. |
| Ogden J, Shaw A, Zander L Women's memories of home birth Part 2: Deciding on a Homebirth British Journal of Midwifery (1997) 5 (4) 212-215 (NB information on methodology taken from Ogden J, Shaw A, Zander L Part 1 Women's memories of home birth 3-5 years on British Journal of Midwifery (1997) 5 (4) 208-211 because it is only included in that part of the three linked articles). | Yes: Explore the factors involved in deciding to have a home birth. | Yes | Yes: interviews in women's own home | Yes, purposive - GPs asked to identify women who had a home birth 3-5 years previously. | Yes, interviews using key research questions. | No; no discussion and identity of interviewer not stated in paper. (Likely to have been research nurse as in other Ogden et al study). | No; ethical review is not mentioned. | Unknown; thematic analysis is described briefly, but not insufficient information to make a judgement. | Yes; there is a summary of themes and categories in a table, but there is no statement about the relationship of themes (synthesis). | Yes, valuable to (1) as the paper presents data specific to choosing home birth. Transferability (2) limited to home birth. |
| Ogden J, Shaw A, Zander L Women's memories of home birth Part 3: A decision with a lasting effect British Journal of Midwifery (1997) 5 (4) 216-218 (information on methodology from Part 1 as above) | Yes: Identify the impact of home birth on women | Yes | Yes: as above (same source) | Yes: as above (same source) | Yes: as above (same source) | No: as above (same source) | No: as above (same source) | Unknown: as above (same source) | Yes: as above (same source) | Yes, as above (same source) |
| Pitchforth E, van Teijlingen E, Watson V, et al. "Choice" and place of delivery: a qualitative study of women in remote and rural Scotland. Quality & Safety in Health Care 2009;18 (1):42-48 doi: 10.1136/qshc.2007.023572. | Yes - 'To explore women’s perceptions of ‘‘choice’’ of place of delivery in remote and rural areas where different models of maternity services are available' (Abstract) and to inform an Discrete Choice Experiment study. | Yes | yes - focus groups with women who had used services | Yes; purposive sampling of women who had used or were using mat services. Mainly PN; 4 pregnant at time of FG. Range of time since last Intrapartum care 4 weeks -7 years | Yes; topic guide and researcher led. Used scenarios that represented OU, MLU [FMU] and home birth to prompt discussions for DCE purposes | Unknown. There is mention of Female 'facilitators' on team -no more information. | Yes: REC approval not needed - MREC applied for but considered to be audit. Paper describes appropriate standard of ethical practice. | Yes: Inductive thematic analysis familiarisation, coding, inter-rater reliability checks, revision of themes in response to new data. | Yes; narrative overview and subthemes and supporting data is evident. | Yes Valuable contribution (1) however transferability (2) limited to other Remote and Rural settings. |
| Pitchforth E, Watson V, Tucker J, et al. Models of intrapartum care and women's trade-offs in remote and rural Scotland: a mixed-methods study. BJOG: An International Journal of Obstetrics & Gynaecology 2008;115 (5):560-9. | To explore women’s preferences for, and trade-offs between, key attributes of intrapartum care models' (abstract). | Yes (to inform DCE study) | Yes; focus groups were held to further explore findings from a questionnaire which had asked questions about women's preferences in relation to attributes of different birth place options/models of care. | Yes; participants were recruited from parent and toddler groups in the eight areas included in the overall study (selected on the basis of having remote and rural services). | Yes; DCE options used as a basis for focus group discussions. | Unknown; not discussed within paper but appears from analysis discussion that authors facilitated focus groups. | Yes: REC approval not needed - MREC applied for but considered to be audit. Paper describes appropriate standard of ethical practice | Yes; familiarisation, coding and constant comparison thematic analysis discussed. Modified by team, suggesting rigour checks were in place. | Yes; focus group findings and categories presented separately and integrated with overall study. | Yes - valuable contribution, particularly in relation to revealed preference (1). Transferability (2) limited to remote and rural areas, as key issues were affected by geography and travel times. |
| Shaw R, Kitzinger C. Calls to a home birth helpline: empowerment in childbirth. Social Science & Medicine 2005;61 (11):2374-83. | Yes: to document the obstacles women encounter in trying to exercise their right to choose to give birth at home | Yes | Yes | Yes; callers to the Help Line were recruited following informed consent conversation. Authors acknowledge this is a self-selecting sample, so the issues should be considered indicative rather than representative. | Yes; naturalistic approach drawing on callers and call handlers' conversation. Explicit feminist perspective may have influenced the framing of questions and responses. Some callers called more than once, and were encouraged to do so, suggesting a supportive relationship had developed. | No; however the research is secondary analysis of transcripts, so there is no direct relationship between researchers and participants. (It may still have been useful to do member checks for content/construct reliability). | Unknown: the authors note that they followed BSA and BPS guidance, and asked Home birth helpline for approval, but it does not appear that an application for independent ethical review of the study was made. | Unknown; content and thematic analysis are reported; there is no information about tests of rigour or reliability of analysis. | Yes; content analysis of reasons for calls and then themes emerging within these. | Unknown (1) replicates difficulties women have experienced in relation to home birth already in literature but with women's perspective more clearly explained and (2) already self-selecting sample and some clearly high risk although this data can be excluded from the review. |
| Stapleton H, Kirkham M, Thomas G. Qualitative study of evidence based leaflets in maternity care. British Medical Journal 2002;324 (7338):639- doi: 10.1136/bmj.324.7338.639. | Yes: To examine the use of evidence based leaflets on informed choice in maternity services | yes | Yes; RCT (intervention = provision of informed choice leaflets) with mixed methods qualitative study | Yes; purposive and convenience sampling of women attending clinics and willing to be observed/interviewed | Yes; but all related to informed choice | No; the researchers' backgrounds are described however. | yes; the study received local ethics approval | No; scant details only, with little information about analysis or rigour | No; findings are presented with onus on health professional views and experiences; women's views appear to be secondary. | Unknown  1. Minimal contribution to literature (few quotes relate to choice of place of birth) 2. Transferability compromised as the leaflets which were investigated are not routinely used in practice, but still identifies general issues of information provision. |
| Tinkler A, Quinney D. Team midwifery: the influence of the midwife-woman relationship on women's experiences and perceptions of maternity care. Journal of Advanced Nursing 1998;28(1):30-5. | Yes: To explore women's experiences of team maternity care, including being informed and making decisions. | yes | Yes: Evaluation study with women receiving team midwifery care and women receiving usual care. | yes; purposive selection of women receiving team midwifery care and also a control group who had usual care | Yes; interviews and focus groups | No; this is not discussed | Yes; local ethical approval gained and ethical issues discussed | No - very minimal information suggesting thematic or content analysis. No discussion of rigour. | Yes; these are organised in relation of quality of relationship with caregiver, which was identified as a central theme. | Yes: (1) confirmatory (continuity of care supported choice) (2) data is transferable but caution in ensuring that quotes are allocated carefully to women receiving/not receiving pilot team midwifery care. Also only a small section on choice of place of birth |
| Walsh DJ. 'Nesting' and 'Matrescence' as distinctive features of a free-standing birth centre in the UK. Midwifery 2006; 22 (3):228-39. | Yes: to explore the culture, beliefs, values, customs and practices around the birth process within an FSBC.[Freestanding birth centre/FMU] | yes | Yes; ethnographic method consistent with study question/aims. | Yes; opportunistic recruitment of women using FMU and willing to be observed/interviewed. | Yes: participant observation; good amount of data, field notes, and interviews with staff and women. | Unknown; this is not discussed, but is demonstrated within the paper. | Yes; local (LREC) ethical approval gained and access negotiated | Yes; full discussion of thematic analysis, categorisation, inductive theory development and theoretical reflexivity | Yes. Findings are presented with data and discussed in relation to literature | Yes (1) useful contribution as little evidence about why women opt for FMU specifically, and (2) transferability likely limited to other FMU settings |
