# Supplementary material for: What influences birth place preferences, choices and decision-making amongst healthy women with straightforward pregnancies in the UK? A qualitative evidence synthesis using a ‘best fit’ framework approach
Source: BMC Pregnancy Childbirth. 2017 Mar 31;17:103. doi: 10.1186/s12884-017-1279-7 (PMC5374625; doi:10.1186/s12884-017-1279-7)
Supplement: Supplementary file 3 — Examples of data and evidence to support a-priori themes (Tables A and B). (DOCX 50 kb) [file 12884_2017_1279_MOESM3_ESM.docx]

**Additional file 3: Examples of data and evidence to support a-priori themes (Table A) and new inductive sub-themes within a-priori theme ‘Antenatal Care’ (Table B)**

Legend

Text in italics within inverted commas= quotes by women participants from included papers.

Text in normal font within inverted commas=quotes from text within included papers (author commentary or summary).

**Table A**: **Examples of data and evidence to support a-priori themes 1-5 (data examples drawn from full framework analysis)**

| **Theme 1 : Information, Knowledge and Empowerment (see Figure 1)** | |  |
| --- | --- | --- |
| Summary of sub-themes and depth of evidence in papers. | | |
| **1.1 (a) Sub theme: Knowledge of right to choose/no choice** | ***Sub theme: examples of quotes from framework analysis*** | **Source** |
| Data in included papers suggested that women did not often feel they were given a choice of place of birth or were only given choices between hospital OU settings. Clear information was not routinely provided. | ‘Many women did not consider they had made a choice or were unaware that a choice was available’ | Houghton et al (2008) p.10 |
|  | *‘I said I would have liked a home birth … “I don’t agree with a home birth” GP said … he didn’t even agree with the birth centre … he thinks that by having your baby there if anything goes wrong you’re then putting not just my life in danger but the baby’s life as well … Well I thought have I got a choice?’* | Jomeen (2007) p.487 |
|  | ‘A notable finding from the focus group was that most women perceived that they had ‘‘no choice’’ in deciding where to deliver, although some did report ‘‘genuine choice.” | Pitchforth et al (2009) p.44 |
|  | *Oh it’s only been a choice of hospital; I can’t even remember being asked the question whether I wanted a home birth. I was given the choice of two hospitals, basically [hospital 1 or 2], but again I suppose I was familiar with 1, I’d had a baby there I was more than happy with the care that I got there so I saw no reason to go and change to a different hospital’* | Madi and Crow (2003) p.332 |
| **1.1 (b) Sub-theme: The right to choose: Being given a choice** |  |  |
| Good support for the proposition that information was important to women within the model; being given choice or provided with information and being empowered to make a choice was important to women. | ‘All participants agreed that the provision of choice in birth place was important’. | Houghton et al (2008) p.9 |
|  | *‘There’s a growing number of home births, I think it is quite a lot of, you know, word of mouth, and people saying it is an option and people not realising it is an option’.* | Houghton et al (2008) p.11 |
|  | *‘I was quite determined I wanted a homebirth. It is just what suited me, what suited my lifestyle...It feels like an achievement that I have done...its standing up for a choice’.* | Ogden et al (1997) Part 2 p.213 |
| **1.2 (a) Sub theme: Information seeking** |  |  |
| Women described asking for information, but receiving limited information. In some disconfirming cases, women did find or receive information, and this was linked to a sense of empowerment (see ‘empowerment’ sub theme below). | *‘I mean you only ever know as much as anybody ever tells you or you bother to ﬁnd out. I am sure there’s lots more information if I can be bothered to ﬁnd out and things, you know, I can get and do. But I mean, I think you only, I personally only asked as much information as I particularly want to know and once I’ve got all the information I particularly need then I’m happy, so, you know, I’m happy with the package that I’ve got’.* | Madi and Crow (2003) p.333 |
|  | ‘Several women also described other sources  of information they had used…: *'Statistics have actually shown...that if you have a homebirth and it goes wrong you can get into emergency just as quickly as you can from the labour ward'.* | Ogden et al (1997) Part 2  p.214 |
| **1.2 (b) Sub-theme: Lack of information** |  |  |
| Included papers consistently report that women experienced a lack of information about birth in different settings.  Women felt they had to look for information themselves, or that midwives might go through the motions of sharing information, without exploring whether it is of interest to women. | *‘You do have to ask about things, and even when I said I want a home birth, I already knew the process because I had spoken to my neighbour about it, but there was nothing given to me. You have to go looking for it or ask for the information yourself deﬁnitely’.* | Madi and Crow (2003) p.332 |
|  | ‘Researcher: “And were you given this leaflet?” [researcher shows woman the Informed Choice leaflet on place of birth]  Woman: *“Oh yes. I had that one but she [midwife] never discussed it with me . . . I thought she would ask me at the next visit if I’d read it but she never did so I just dropped it really. It wasn’t that important . . . I’m quite happy with [hospital X]. I don’t know why she bothered giving it me. I did wonder that.”’* | Stapleton et al (2002) p.3 |
|  | *‘I* *would have liked to have been really clear about what my personal choices were, based on my situation… initially, when you are pregnant, you should be given a pack that gives you all the choices in your area’* | Barber et al (2006) |
| **1.3 Sub theme: Empowerment** |  |  |
| Receiving support for home birth from a midwife provided a sense of empowerment. | *‘She (the midwife) was all for home births… I felt really empowered just going to that class... she told us how to breathe, how to get through.’* | McCutcheon and Brown (2012) |
|  | ‘...*the midwife kept giving me, telling me bits of information to tell him...to have the baby at home, because they said there was...less chance of the baby catching any sort of infections if it is born at home*.' | Ogden et al (1997) (Part 2) p.214 |

| **Theme 2**  **Preferences (aspects of services and attributes that contribute to preferences or inclination towards birth settings) – see Figure 1** | | |
| --- | --- | --- |
| Summary of sub-themes and depth of evidence in papers | **Sub theme: examples of quotes from framework analysis** | **Source** |
| **2.1 (a) Sub-theme Preference for or inclination towards hospital OU** |  |  |
| Strong support: consistent reports across numerous papers help establish that some women describe preferring or feeling more inclined towards the hospital OU setting.  Several codes contributed to this sub-theme, including safety, comfort, confidence, convenience.  Key sub-themes included the belief that hospital is ‘safest’, that women feel ‘confident’ in the hospital.  The availability of medical staff and facilities underpins that confidence, and means that women do not have to consider transferring during labour. | **Safety** |  |
|  | ‘Key themes that emerged from the women’s focus groups around decision making included the hospital being regarded as safest, especially with the first pregnancy, but that conflicting advice from different health professionals made the decision-making process difficult.’ | Barber et al (2006) p.612 |
|  | ‘They all chose hospital birth:  “*Because hospital has medical cover and equipment*” “...*it is safer in hospital*”’ | Cheung (2002) p.207 |
|  | *‘In deciding where to have the baby, I guess I was pretty determined I’d have it in hospital [OU]. Both my sister and my mother had problems during birth…so it made sense given the experiences of people close to me that I’d like to be somewhere with good medical care on hand, if something goes wrong’*. | Coxon et al (2014) p.58 |
|  | **Pain relief** |  |
|  | ‘Some felt it was important to have all types of pain relief available, particularly for ﬁrst-time mothers and knowing it was there provided general reassurance.    “*I think if you haven’t had a child it’s important to know it’s there, isn’t it?*”  “*It’s like a safety net. The knowledge of it being there, even if you don’t want it. It’s there and that’s like a, takes away fears*”.’ | Pitchforth et al (2008) p.566 |
|  | **Medical support and facilities** |  |
|  | *‘I would feel a lot more comfortable being in a medical sort of environment knowing that if anything went wrong, everything is there for me rather than at home, where ok you might feel more comfortable being at home, but I would still have quite a few reservations about being at home. I’d feel a lot happier at the hospital’* | Houghton et al (2008) p.8 |
|  | *‘Although midwives are kind and caring, you feel more confident if you know that a doctor is around, just in case there’s an emergency.’* | Lavender and Chapple (2005) p.51 |
|  | **Avoid transfer** |  |
|  | ‘Characteristics identified by the hospital birth [focus] group [included]:  …Specialist medical facilities to deal with any problems which might arise for mother or child are available on site, precluding the need to transfer during labour’. | Longworth et al (2001) p.406 |
| **2.1(b) Sub theme**  **Preferring to avoid OU or negative about OU setting** |  |  |
| Included papers contained sufficient evidence to generate understanding about why some women prefer to avoid the hospital OU setting; these include phobia about hospitals, previous poor experience of impersonal care in OU and likelihood of interventions or being persuaded to have these.  **Remote and rural settings**  For some, the distance needed to travel to get to hospital OU was problematic – this was found when women lived in remote or rural settings where they or their families would need to travel long distances, sometimes by airplane or boat, to hospital OUs. | **Phobia** |  |
|  | ‘For the following participant her overriding fear of hospital made it impossible for her to consider anything but a home birth:  *“I got a thing about hospitals and doctors — it’s my phobia. I hate it”.* | Andrews (2004) p.519 |
|  | **Rest** |  |
|  | *‘I just wanted to go home straight away and I didn’t want to have a shower or a bath in hospital and I wasn’t ill and couldn’t rest because you’re in a ward with everybody.’* | Andrews (2004) p.520 |
|  | **Distance** |  |
|  | ‘So, when you were deciding, can you say what the biggest priority was for you?  “*I think distance because there’s nae [not] really, like my partner’s mother, she’s disabled … there’s no folk in my family drive, so getting through to [referral consultant unit] is a bit of a hassle*”.’ | Pitchforth et al (2009) p.46 |
|  | **Interventions** |  |
|  | “…*a close friend has given birth in hospital [OU] because she had to be induced, and the whole procedure [...] it just seems kind of more forced on you and more [...] scary, rather than just doing it at your own pace and dealing with it and the pain and everything that’s happening at that time yourself. So ideally I’d stay [home] as long as possible and then go to the FMU*.” | Coxon et al (2014) p.62 |
|  | **Impersonal care** |  |
|  | *‘It was totally impersonal. I was another body. I was just number eight in the night or whatever it was ... So that was one of my main reasons for choosing the DOMINO.'* | Mansion and Maguire (1998) p.665 |
|  | ‘*Things happen like they left you to it for ages and ages-because they didn't have a bed, which was fine, and then as soon as they had space in the labour ward they dragged you up and told you that you would have to be monitored all the time and weren't allowed to move.'* | Ogden et al (1997) p.213 (Part 2) |
| **2.2 (a) Sub-theme: Preference for or willingness to plan birth at home** |  |  |
| Papers which considered women’s alternatives to hospital predominantly reported a choice between ‘hospital’ and ‘home’.  Key findings included feeling comfortable or relaxed in own environment, feeling in control during birth, having a known midwife/carer, and knowing others who have had a home birth.  Data which contributed this sub-theme came from numerous papers, and was consistent across these. | **Feeling more relaxed** |  |
|  | *‘It would feel more normal and I could say well, I fancy a jam sandwich now and watch tele and do what I wanted — It was about being able to do more of what I wanted to do.’* | Andrews (2004) p.521 |
|  | *‘I expected it to be quite, quite sort of calm, the setting, quite relaxed and just a nice calm environment really.’* | Andrews (2004) p.521 |
|  | **Feeling in control** |  |
|  | *‘…you’ve got control over your environment, you can decide what position you’re in, whether you need something to eat or a bath or a scented candle or, you know, you might want none of those things…’* | Coxon et al (2014) p.62 |
|  | *‘I felt in control… you know on the floor being on, over a ball and being over the arm of a chair, and... just listening to my body.’* | McCutcheon and Brown (2012) p.10 |
|  | *‘You are very much in control because it’s your home—it’s your territory.’* | Shaw and Kitzinger (2005) p.2377 |
|  | **Known carer** |  |
|  | *‘More for me rather than just one body in a ward - more about me because my midwife will have had more information about me.’* | Mansion and Maguire (1998) p.4 |
|  | *‘I did write a birth plan* [for home birth], *but I didn’t need to because it was all in her head… because we discussed it all… She knew exactly where I was coming from.’* | McCutcheon and Brown (2012) p.9 |
|  | **Feeling safe** |  |
|  | ‘*Birthwise I have always thought that it* [home birth] *was a very safe option. You have a very skilled midwife, or probably two skilled midwives, possibly a student midwife as well at the birth, possibly a GP. For many births that take place in hospital, you don't have that skilled attendance. The house was easily accessible for emergency services and not very far from the hospital.'* | Ogden et al 1997a Part 2  (p.215) |
|  | ‘Some women in the sample managed anticipated risks in planning their home birth through considering proximity to the hospital to have swift access to medical resources: “*I only live... ten minutes’ drive from the hospital so I felt if I had to go in… we’d take the decision early enough to get there*.” | McCutcheon and Brown (2012) p.8 |
| **2.2 (b) Sub theme: Preferring not to plan birth at home** |  |  |
| The included papers contained clear indications that some women preferred hospital and would not wish to give birth at home. Whilst the most frequent explanation was that home was not considered as safe as hospital, women also actively wanted to avoid home birth. | **Don’t feel home is safe setting for birth** |  |
|  | ‘Even the second time Scottish mothers resisted the comfort, familiarity and apparent safety of home birth:  “*Hospital for me and it gives me conﬁdence, you know. If anything goes wrong, help is there. And being at home doesn’t hold any particular attractions for me and I do not, ...I certainly had no conﬁdence at home. It’s no doubt*”.’ | Cheung (2002) p.207 |
|  | *‘I’ve never thought of giving birth to a child at home. There are too many fears*.’ | Cheung (2002) p.207 |
|  | **Home is the ‘wrong place’ for birth** |  |
|  | ‘Several women thought it was important to separate their birth experience from their home environment as a way of protecting themselves or their family from the birth itself or the memory of it. These women viewed birth as a potentially unpleasant, upsetting or embarrassing situation:  “*I always felt that with a home birth, if it’s not as comfortable a process as you would like it to be you’re kind of walking into the same room, you know if it’s in your house you’d remember it”.’* | Houghton et al (2008) p.8 |
|  | **Associating home birth with ‘new age’ lifestyle** |  |
|  | *‘If someone wanted to do it (birth at home), I’d think they were a new age hippy.’* | Houghton et al (2008) p.11 |
| **2.3 (a) Sub theme: preference for or willingness to plan birth in AMU** |  |  |
| Whilst six papers do include data from women who had option of AMU birth, most report data on home and hospital and provide little data on choice or preference for AMU.  Where preference for AMU is discussed, findings suggest that philosophy and attributes of the setting are important, and this is similar to the home birth data. AMUs were preferred due to the opportunity for ‘natural birth’ in a ‘familiar environment’ where partners could stay. Proximity to OU is also thought to be important | **Familiar environment** |  |
|  | *‘I went to see the birth centre, and it was beautiful. I projected myself with Mick immediately in that situation, and it was perfect because I wanted to go in the pool. (It) is a more familiar environment than hospital. I could see myself there immediately.’* | Newburn (2012) p.64 |
|  | Many women wanted to use the birth centre because: ‘*It’s all natural*’ or *‘They do it as natural as possible’*. Their motives varied from wanting to fully experience the sensation of birth to wanting to avoid the effects of medical interventions:  *‘I wanted a natural birth, I wanted to feel it.’* | Newburn (2012) p.64 |
|  | ‘The separation from the labour ward yet close proximity on the same site gave women a sense of security. They felt that if they needed to transfer for an epidural or medical care they could do so quickly, so they felt safe’*.* | Newburn (2012) p.65 |
| **2.3 (b) Sub theme: preferring not to plan birth in AMU** |  |  |
| As few papers explored choice of or preference for AMU, there was little evidence about the reasons women may prefer not to go to AMU The idea that an AMU is ‘not clinical enough’ was put forward by two women during postnatal interviews. | **Not clinical enough (AMU)** |  |
|  | ‘*There’s no equipment on show or anything, that’d just put me off a bit. I’d want there to be a little bit of equipment’* (Alison). | Houghton et al (2008) p.8 |
|  | ‘*Oh no, that would frighten me that. Big bath*. (laughs). *Birthing pool. No, there’s no machines. So, I’d feel like I was going on me happy hols in that* (laughs) *not going in to give birth*’ (Joely). | Houghton et al (2008) p.8 |
|  | **Reduced access to epidural** |  |
|  | *‘I want to go to the midwife-led unit as I’ve heard it’s really relaxed but my mate went there and had to wait ages for an epidural because the doctor was doing one on the main delivery suite.’* | Lavender and Chapple (2005)p.51 |
| **2.4 (a) Sub theme: preference for or willingness to plan birth in FMU** |  |  |
| The synthesis included seven papers which contained data about women’s reasons for preferring or considering planned FMU births.  Key elements of this theme were similar to those for preferring, considering or being inclined towards home birth: known carer, having family present, and birth in a homelike or familiar environment. | **Baby and family friendly environment** |  |
|  | ‘One woman commented on the fact that she was greeted at the door by a staff member holding a baby. She concluded that this was a baby- friendly environment.’ | Walsh (2006) p.231 |
|  | ‘*I think that you should try to include the whole family in this. It would be impossible really to drag other kids up and down to* [referral consultant unit] *the whole time’* | Pitchforth et al (2009)p.46 |
|  | **Homely environment** |  |
|  | *‘The* [FMU] *is just like being at home … I just liked the whole atmosphere, the fact that it was so relaxed, and I liked the birthing pool.’* | Jomeen (2007) p.488 |
|  | **Known carer** |  |
|  | ‘*I chose* (FMU) *because I would like the midwife I have had all through my pregnancy to be the midwife to deliver my baby.’* | Emslie et al (1999) p.200 |
|  | **Relationship with midwives** |  |
|  | ‘I just think that midwives tend to be sympathetic and have more of an open ear. Consultants tend to think more on outcomes than on the experience.’ | Pitchforth et al (2009) p.47 |
| **2.4 (b) Sub-theme: preferring not to plan birth in an FMU** |  |  |
| Key elements which affected preference for or willingness to plan birth in FMU were anxiety about transfer from the FMU into an OU, and a sense that there were safety concerns, or that women would feel blamed or responsible if they opted for FMU and something went wrong.  Three papers contained qualitative data about women’s reasons for preferring not to give birth in FMUs | **Safety and transfer concerns** |  |
|  | *‘I feel that* [FMU] *is unsafe, because if you or*  *the baby was to suffer difﬁculties during or after birth there is no adequate facilities, so therefore you would have to endure a very distressful journey to the Host unit*’. | Watts et al (2003) p.110 |
|  | **Feeling responsible** |  |
|  | ‘*If you know that there is no consultant there and no pain relief, …and you choose to go there [FMU] and you end up in an ambulance going to [OU] on a blue light then it’s all your own fault you could sign a form, if anything goes wrong then the women take it on themselves*.’ | Watts et al (2003) p.111 |

| **Theme 3** **Influences: Women’s previous birth experiences – see Figure 1** | | |
| --- | --- | --- |
| **3.1 (a)Sub theme: previous birth experience in Hospital OU** | **Examples of quotes from framework analysis** | **Source** |
| A consistent finding was that women who had previously given birth in hospital OU settings would give birth in hospital again, even if they had previously planned birth in non-OU settings. There was some disconfirming data which suggested that poor hospital birth experience would lead women to plan future birth in a non-hospital birth setting.  Four papers contained data about the influence of hospital (OU) birth on current or future births. | **Would plan birth in OU in future** |  |
|  | ‘*Is that what I said last time.. .?* [Referring to preference for home birth in antenatal interview] *I think I’ve changed my mind. I would deﬁnitely go into hospital. I felt safe there. I think I’d be more stressed having it at home.. . I don’t think I would ever consider a home birth... I don’t think I’m brave enough’*. (Sarah, following birth of ﬁrst baby, low-risk pregnancy) | Coxon et al (2015) p.4 |
|  | ‘*Was I naıve? I don’t think so. But I was quite .. . optimistic*  *about birth, you know, ... but yes, it was .. . altogether more unpleasant .. . it was worse than I thought*. (Alison, ﬁrst baby, low-risk pregnancy) | Coxon et al (2015) p.4 |
|  | ‘Her subsequent birth experience in hospital affected her self-confidence and her decision on where to give birth in this pregnancy: “*If I hadn’t had an epidural last time, I think there’s no doubt I would have thought really hard about having a home birth this time*” (Lorna). | Houghton et al (2008) p.10 |
|  | *‘I think I’d probably still go for a hospital birth, even though I delivered with just gas and air this time and so that’s quite possible at home... both hospital births for me have worked out for us as a family so I’d probably be inclined to go that way again*’ (Heather, PN). | Houghton et al (2008) p.13 |
|  | **Would plan birth in non-hospital setting in future** |  |
|  | ‘Whilst discussing their reasons for choosing a home birth, the women reflected on previous births in hospital. Some of the women remembered feeling nervous, afraid, ill informed, powerless and intimidated in the hospital environment: “*You feel as though you’re interfering in your own birth*”.’ | Andrews et al (2004) p.520 |
| **3.1 (b) Sub-theme: previous birth experience at home** |  |  |
| This theme was well supported. Eight papers contained data about the influence of previous home birth experiences; these suggested that successfully giving birth at home meant women felt more confident about birth at home or in FMUs in future.  However, some women who encountered problems during birth in non-hospital settings reported that they would plan birth in hospital in future. Women also reported feeling disappointed after unsuccessful attempts to give birth at home. | **Previous home birth: would plan birth at home or in non-OU setting in future** |  |
|  | ‘*I think I would* [give birth at home again in the future] *yeah. Unless there were any complications .. . I think I probably would*.’ (Holly, second baby, low- risk pregnancy) | Coxon et al (2015), p.5 |
|  | ‘*After my second child I was very negative but* [this home birth] *being such a nice calm experience... If I was to get pregnant this would make me do it again*.' | Ogden et al (1997b) Part 3 p.217 |
|  | **Confidence in midwives’ skills** |  |
|  | ‘*I’m not worried about having a special care baby unit. I have had a home birth before and know the midwives can deal with problems. The chances of anything going wrong are so small anyway’* (Planning FMU birth this time). | Lavender and Chapple (2005) p.51 |
|  | **Wouldn’t plan birth at home in future** |  |
|  | ‘One woman who had had a difficult [home] birth said: “*It did make me nervous about having* [this baby]. *I decided that I would have* [this baby] *in hospital*”.’ | Ogden et al (1997) Part 3 p.217 |
|  | **Feeling disappointed** |  |
|  | ‘One participant stated that the effect of being unable to fulfil her hopes of a home birth led to self-criticism: “*My ideals of the natural birth… my body didn’t seem capable of doing that*.” | McCutcheon and Brown (2012) p.8 |
| **3.1 (c) Sub theme: previous birth experience in FMU/AMU** |  |  |
| Four papers included data from women whose previous FMU birth experiences influenced current choices; these papers suggested that women who gave birth in FMUs benefited from good care and received good postnatal support, and would plan to give birth in FMUs again.  Whilst included papers did cover AMU options, none of the reported data referred to how experience of birth in AMU influenced current or future birth preferences. | **Would plan birth in FMU again** |  |
|  | ‘*I would* [go to the FMU in a future pregnancy] *because of the treatment that we had there…everyone was so good*’. | Coxon et al (2015) p.5 |
|  | ‘I usually asked the women I interviewed why they had chosen [FMU]. For some, their own childbirth history inﬂuenced their decisions. Seven had had babies in the unit before, and wanted to return there’. | Walsh (2006) p.230 |
|  | ‘*In* [referral consultant unit] *you have to buzz for them to notice, whereas here* [FMU] *they have the time to notice before you would have to say. They pop in and out the whole time basically*.’ | Pitchforth et al (2009) p.45 |
| **Theme 4**  **Influences: Other experiences which have influenced women’s birth place preferences – see Figure 1** | | |
| **4.1 Sub theme: Views of friends and family** |  | **Source** |
| Views of friends and family and birth stories from other women were an important influence on birthplace decisions; each reflected or contained ‘cultural narratives’ about birth.  The influence of family and friend views was most evident in papers which discussed home birth. When women planned hospital birth, they did not feel a need to seek the views of friends or family. This observation was linked to the ‘cultural norm’ sub- theme. | **Views of family and friends: home birth** |  |
|  | ‘*When I said to* [husband] *about home birth he was against having it in the house at first. I said if we’re gonna do it we got to do it wholly together, we can’t do it all of me and some of you, we got to do it 100% each.*’ | Andrews et al (2004)p.520 |
|  | ‘*In fact my husband was quite keen, having had the experience of homebirth in the country, to have the first baby at home, but I was less confident*.' | Ogden et al (1997) Part 2 p.214 |
|  | ‘*My friends weren't keen...because they were thinking predominantly about my health and the health of the baby and they thought that in hospital if anything goes wrong I would be completely sup- ported by modern technology*.' | Ogden et al (1997) Part 2 p.214 |
|  | *‘To be honest with you the main reason I’m calling is because my friends and my mum and just about everybody else I meet apart from my husband look at me in complete horror when I tell them I want a home birth. I was in tears last night because I had a talk with one of my friends yesterday who basically said to me ‘look you know I appreciate you’re doing it but I couldn’t put my child’s health at risk like that’*. (Ursula 13) | Shaw and Kitzinger (2005) p.2378 |
|  | ‘*My brother had … well his wife had a baby at home and the baby died … and I think that affects…that sort of affects the family for a long time, you know, anyone in the family who was involved with that or remembers that, you can’t*, [home birth is] *just a no-no for us*. (Patsy, multiparous, healthy pregnancy and straightforward obstetric history, planned OU birth) | (Coxon et al 2014)  p.59 |
|  | **Views of family and friends – hospital birth** |  |
|  | ‘…most women who were planning a hospital birth had not discussed the place of delivery with their partners:  “*We never really discussed it because we knew when* [name of hospital] *closed down, we just thought oh well, next time we have a baby it will be at* [name of hospital]. *So, um, yes, it wasn’t something we discussed, it was just, that was where we were going to go”*.’ (Olebogeng, hospital) | Madi and Crow (2003) p.333 |
| **4.2 Sub theme: Stories of other women** |  |  |
| Other women’s stories are important for affirming ‘non-OU’ choices (again, this is linked to the cultural norms theme). Four papers contained references to stories from other women: | ‘…[friend] *in the chip shop his wife had a baby at home and he said it was better. We always used to talk about it*.’ | Andrews et al (2004) p.520 |
|  | *'I had quite a few women friends who had had babies at home...and there were certainly a few people who had had wonderful experiences of having their babies at home...So you...heard about their birth experiences and they were all very positive about it.*' | Ogden et al (1997) Part 2 p.213 |
| **4.3 Sub-theme: Cultural norms or cultural narratives** |  |  |
| This theme was well supported by data from included papers. Seven papers specifically referred to normative ideas, and each identified hospital (OU) birth as what was considered to be ‘normal’, in UK and in other countries. | **Hospital birth as culturally normal** |  |
|  | ‘*Well basically I think it’s just what I’ve known. That’s what my mother did, all my family members have had their babies in the hospital, and I just think it’s a safer place, in case of any kind of emergency it’s better to be at the hospital where there’s a doctor close by.*  *…I think it’s culture as well, for me. It’s just where we’re from. You only have a baby at home if you can’t make it to the hospital, and even when that happens people are* […] *almost ashamed to say. They’ll still say, ‘Oh yeah, we went to the hospital*.’ | Coxon et al (2014) p.59 |
|  | *‘I just thought, what? What a strange thing to ask. Because for me, being* [from a Nordic country], *when you want to have babies, you go to the hospital, just like if you want to have an operation you go to hospital!’* | Coxon et al (2014) p.59 |
|  | ‘…another participant identified a cultural narrative that women who have a home birth are unstable for taking perceived risks in giving birth at home: “*Culturally a lot of people think that you’re risking life and limb… and a bit mad, a bit crazy*.”’ | McCutcheon and Brown (2012)p.6 |
| **4.4 Sub-theme: Home birth service not guaranteed** |  |  |
| Women reported that home birth services were not guaranteed, or that staff might not be available when they went into labour. | ‘*You’d ring up in labour and be told there were no midwives available and so you’d have to come in and that would have been my disaster scenario.* | McCutcheon and Brown (2012), p.6 |
|  | ‘Several callers reported that they had asked for midwifery cover for a home birth and been denied it because of staff shortages, or simply because, as one doctor is reported to have said, ‘‘*we don’t do home births here*’’*.’* | Shaw and Kitzinger (2005) p.2378 |
| **Theme 5**  **Beliefs and perceptions regarding birth risks in general or in specific settings – see Figure 1** | | |
| **5.1 Sub theme: Safety beliefs** |  | **Source** |
| Substantial evidence to support the notion that hospital is considered the ‘safest’ place for birth, at least compared with home.  Women who were considering home birth also reflected on the safety of home births. One paper included data proposing that home birth was the safest setting, and others argued that homebirth was beneficial for women and babies (see ‘preferring home’ subtheme). | **Hospital OU is safer** |  |
|  | ‘*From the safety point of view, hospital is a better place ... I still feel* [home delivery] *is impossible*.’ | Cheung (2002) p.207 |
|  | *‘I would deﬁnitely go into hospital. I felt safe there’.* | Coxon et al (2015)p.4 |
|  | ‘*I think it would be safer in hospital. I mean if you’ve got the midwives at home and you’ve got the midwives in the hospital, you could say you’ve got the same thing, but as I say, you just don’t know what else you’re going to have to use’.* | Houghton et al (2008)p.6 |
|  | ‘*She was our first baby so we obviously just thought it was better to go to hospital, much safer than having it at home*.' | Ogden et al (1998)p.341 |
|  | ‘Participants associated [OU] with covering every eventuality, “*having everything there*” or, as one woman described, being the “*ultimate safety net*”. | Pitchforth et al (2008) p.564 |
|  | **Home birth safety** |  |
|  | ‘*People like my mum panicking about having a home birth, it’s kind of well, like things go wrong in hospital too.*’ | Andrews et al (2004) p.520 |
|  | ‘*She* [the midwife] *kind of reassured me a bit on a few things, you know, what happens in an emergency and that kind of thing… In the end we came up with the decision that it might be even safer to have the baby at home with me having such a quick labour* (last time).’ | Houghton et al (2008) p.7 |
| **5.2 Sub theme: Risk beliefs** |  |  |
| Data from included papers provided good support for the inclusion of ‘risk beliefs’ as an a-priori theme; risk-based reasoning was used to deter women from giving birth in settings other than hospital OU, or by women themselves to explain why they chose hospital OU over non-OU settings; for this reason, this is also conceptually linked to the sub-theme of cultural norms/narratives. | **Risk-based decision making** |  |
|  | ‘The consultant and the GP were looking for problems, were saying “*You need to come in to hospital in case something goes wrong*” | Barber et al (2006) p.611 |
|  | ‘…he [GP] said, “*Yes, I’m just saying, you know, because you know the chances are … It’s a 40 minute journey* [referring to transfer from FMU to OU during labour]. *Do you want to risk that?” No*! [Laughs] *But yeah, that’s all he really said, but he was right…I’m not risking that, I’m not risking the baby’s life or my life. So … you know, it’s just eliminating all the risks as much as you possibly can”.’* | Coxon et al (2014) p.58 |
|  | ‘*I didn't really want to go into hospital. I have heard a few scary stories which have put me off...but having said that I didn't want to put my baby's life at risk. If anything happened with me not going into hospital, I know I would never have forgiven myself*.’ | Ogden et al (1997a) (Part 2) p.215 |
|  | ‘*I said why can’t I go to the* [FMU]? *And she said Oh! Well this is your ﬁrst child, we really wouldn’t advise it. There won’t be any doctors present and, if anything goes wrong, they will have to take you to the hospital*.’ | Walsh (2006) p.231 |
| **5.3 Sub-theme: Iatrogenic risks (risks posed ‘to’ natural or normal birth’)** |  |  |
| The theme of ‘iatrogenic risk’, or risk posed to ‘normal’ or ‘natural’ birth by interventions, was supported, although this issue was discussed much less frequently than ‘risk’ or ‘safety’, and was exclusively considered a ‘risk’ posed by hospital [OU] settings and one which women plan birth in non-OU settings to avoid. | **Hospital OU births are more likely to involve interventions** |  |
|  | ‘Several women felt the [AMU] would protect them from  the kind of interventions it was difﬁcult to avoid on the labour ward:  “*I wanted to know what on earth it felt like. I knew I wanted to have a go at a normal birth and so I wanted to be somewhere where the temptations were less and where the midwives were into normal birth*”.’ (Alison, ﬁrst born with an epidural) | Newburn (2012) p.64 |
|  | *‘I was quite scared of going into hospital because then you get interventions… they’re kind of timing you, that’s the impression I get*. | McCutcheon and Brown (2012) p.12 |
|  | ‘Callers [to a home birth helpline] reported wanting to avoid medical interventions such as a hospital ‘‘*one in four caesarean rate*’’, (Tanya 31) or foetal electronic monitoring (‘‘*they put you on the monitor—and that’s the way you stay, and you’re in no position to argue because you’re seriously in pain and unless you’ve got someone speaking on your behalf I think you’re trapped*’’ (Alena 15). | Shaw and Kitzinger (2005) p.2377 |
| **5.4 Sub-theme: ‘Care quality’ or impersonal care risks** |  |  |
| Hospital OUs were sometimes perceived to be too busy to give personalised support. This theme was well supported, with in-depth data and quotes from four papers. | ‘*Worst case scenario, going into hospital and not getting any personal treatment, where they come into the room and they don’t even speak to you, they just speak to your partner, and poke you about and start examining you just makes me feel I don’t know... it’s just very invasive*.’ | McCutcheon and Brown (2012)p.7 |
|  | ‘*I just know how easily mistakes are made, and it worries me, that I’m putting my faith and the life of my child in a* [...] *in an overrun crowded hospital full of people giving birth* [...] *yes, does make me a bit nervous.’* | Coxon et al (2014) p.16 |
| **5.5 Sub-theme: Risks of transfer See Theme 2 (2.1a, 24b).** |  |  |

Table B: Theme 6 – Antenatal Care Experiences (see Figure 1).

| A priori-theme 6 Antenatal care  New inductive sub themes: Women’s antenatal experiences: Finding out about choice of place of birth and making the decision |  |
| --- | --- |
|  |  |
| **6:1 (a) Influence of sociocultural context *non-supportive* towards planning birth at home or in FMUs** | **Evidence from included papers (examples drawn from full analysis)** |
| Home birth is considered counter-cultural, hippy, non-normative or alternative (Houghton et al. 2008; Lavender & Chapple 2005; Ogden et al. 1997a; McCutcheon & Brown 2012)  Home is not ‘the right place’ for birth (Houghton et al. 2008; Lavender & Chapple 2005).  Friends and family oppose plan, or concerned about risks to baby’s life (Shaw & Kitzinger 2005).  Women said they need their partners’ support for home birth (but not for planned OU birth) (Andrews 2004a).  Women felt responsible for the outcome if they planned a home birth (Stapleton et al. 2002) | ‘*If someone wanted to do it* (birth at home), *I’d think they were a new age hippy*’ (Lisa, AN). (Houghton et al 2008, p.11)  ‘*Culturally a lot of people think that you’re risking life and limb… and a bit mad, a bit crazy*.’ (McCutcheon and Brown 2012, p.6)  ‘*I always felt that with a home birth, if it’s not as comfortable a process as you would like it to be you’re kind of walking into the same room, you know if it’s in your house you’d remember it*’. (Houghton et al 2008 p.8)  ‘..*my friends and my mum and just about everybody else I meet apart from my husband look at me in complete horror when I tell them I want a home birth*’.(Shaw and Kitzinger p.2378)  ‘… *When I said to* [husband] *about home birth he was against having it in the house at first*... .’ (Andrews 2004 p.520)  ‘[The obstetrician said]…*if anything went wrong it would be my own fault; it would be on my own head. It was horrible. I came home and cried*.’ (Stapleton et al 2002 p.4) |
| **6.1 (b) Influence of sociocultural context supportive towards planning birth at home or in FMUs** |  |
| It helps to know somebody who had given birth at home (Houghton et al. 2008; Ogden et al. 1997a; Madi & Crow 2003) or in an FMU (Walsh 2006).  Home birth: Having a husband/partner/family friends who are supportive of home birth plan (Houghton et al. 2008; Ogden et al. 1997a; Madi & Crow 2003). | ‘*There’s a growing number of home births, I think it is quite a lot of, you know, word of mouth, and people saying it is an option and people not realising it is an option maybe until you meet other people who’ve done that*.’ Houghton et al [2008] p.11/12  ‘*I had spoken to a family friend who had had one out of three daughters at the birth centre* [FMU], *and she said that it was by far the best. She had had her other two at neighbouring hospitals*’. Walsh [2006] p.230  ‘*My husband said to me, whatever you want is ﬁne by me. So, yes, he is quite understanding and just said well, I can’t understand why you want to have it at home, but I’m with you a 100%*’. (Madi and Crow 2003 p.333) |
| **6.2 (a) Influences from womens’ own perspectives: pre-pregnancy, antenatal experiences, own beliefs and previous birth experiences non-supportive to birth at home or FMU.** |  |
| Countering the negativity (Andrews 2004)  Having to be strong or brave (Coxon et al 2015, McCutcheon and Brown 2012, Ogden et al 1997a, Pitchforth et al. 2009 )  Forewarned is forearmed, feeling embattled (Andrews 2004, McCutcheon and Brown 2012, Shaw and Kitzinger 2005)  Concerned about need to transfer or long journey from home (Pitchforth et al. 2009; Watts et al. 2003) or FMU (Emslie et al. 1999; Lavender & Chapple 2005; Watts et al. 2003; Pitchforth et al. 2009; Pitchforth et al. 2008) | ‘*I wasn’t evangelical about it but I felt you almost had to be to counter the negativity coming at you’*. (Andrews 2004 p.520)  *'I was quite determined I wanted a homebirth. It is just what suited me, what suited my lifestyle...It feels like an achievement that I have done...its standing up for a choice*.' (Ogden et al 1997a p.213)  ‘*I was just ringing to see if you can give me a bit of advice, just to make sure. You*  *know, forewarned is forearmed*’ (Shaw and Kitzinger 2005 p.2377)  ‘*I was worried she would be born in the lay-by beside the road. I was really worried about that. It didnae happen, thank goodness, but that was my biggest concern. That was one of my concerns as well if, I knew I had got to go through to* [referral consultant unit] *and I was just worried that I wouldn’t get there*’. [FMU birth] (Pitchforth et al 2009, p.46) |
| **6.2 (b) Influences from womens’ own perspectives: pre pregnancy, antenatal experiences, own beliefs and previous birth experiences *supportive* towards planning birth at home or FMU** |  |
| Having a previous positive birth experiences at home (Andrews 2004; Ogden et al. 1997b) or in FMU  Planning a home birth is empowering (McCutcheon and Brown 2012) | ‘For some, their own childbirth history inﬂuenced their decisions. Seven had had babies in the unit before, and wanted to return there.’ (Walsh, 2006 p.230)  ‘Overall, the participants who gave birth at home reported an immense sense of achievement which remained with them after the birth: “*I think it’s something I carry along with me, chuffed to be able to have her at home. It was a case of being able to do everything that I’d planned*.” (McCutcheon & Brown 2012, p.11) |
| **6.3 (a)**  **Influences arising from services (service provision, staff information provision by healthcare professionals) experienced as *non-supportive* towards planning birth at home/FMU.** |  |
| Option not discussed or conversation ‘blocked’  (Ogden et al. 1997a; Shaw & Kitzinger 2005; Tinkler & Quinney 1998; Houghton et al. 2008; Jomeen 2007; Pitchforth et al. 2009; Lavender & Chapple 2005; Mansion & McGuire 1998; Stapleton et al. 2002; McCutcheon & Brown 2012; Madi & Crow 2003)  Not allowed to have a home birth  (Pitchforth et al. 2009; Shaw & Kitzinger 2005)  Being told you are reckless or selfish  (Coxon et al. 2014; Jomeen 2007; Lavender & Chapple 2005; Stapleton et al. 2002; Ogden et al. 1997a; Shaw & Kitzinger 2005)  Needing to prove or demonstrate eligibility for planning birth in non-OU settings.  (Andrews 2004; Barber et al 2006, Houghton et al. 2008; McCutcheon & Brown 2012; Jomeen 2007) | Did [GP] talk to you very much about where you can deliver or...] ‘*No*’ [I: Who gave you that kind of information then?] ‘*I haven’t really had any of that kind of information*.’ (Jomeen 2007 p,487)  ‘*I just wanted to be at home, but I had a lot of cheek sucking from the midwife*.’ (McCutcheon and Brown 2012 p.6)  Jane: *‘(I was)* *talking to the midwife at my GP’s surgery and she said would you consider a home birth* [Good eye contact, leaning toward midwife,] *I just worry about it in case something happens if the baby’s not breathing or…*’ Midwife: ‘*Yes*’ [silence]. Jane: ‘*Right ok so*...’ [The midwife continues with the booking interview without further reference to birth place.] (Houghton et al p.10, observation data)  ‘*I’ve just been for my ﬁrst midwife’s appointment and we said we’d like to be at home and she said she didn’t allow home births*.’ (Shaw and Kitzinger 2005 p.2378)  ‘…*they’re making me feel so selﬁsh*.’ (Shaw and Kitzinger 2005 p.2378).  *‘I do think you can be bullied into things, particularly if you’re not strong minded about what you want. It’s quite easy for them to bully you, they say things like ‘The baby will die’ if you don’t do so and so*’ (Stapleton et al 2002 p.4)  ‘*I was trying not to get too excited because [midwife] said from the beginning if there’s anything wrong, your blood pressure or anything you’ll be going into hospital*’. (Andrews 2004 p.521)  ‘*The consultant and the GP were looking for problems, were saying “You need to come in to hospital in case something goes wrong”, and I think there is a difference between the consultants and the people that deal with pregnancies that go wrong and midwives services, because they are looking for issues and problems when there aren’t necessarily any there*.’ (Barber et al 2006 p.611) |
| **6.3 (b)**  **Influences arising from services (service provision, staff information provision by healthcare professionals) experienced as supportive towards planning birth at home or in FMU.** |  |
| Being offered a home birth by a midwife.  (Andrews 2004; Madi & Crow 2003; Houghton et al. 2008)  Trusting the midwife, and her skills (home, AMU, FMU)  (McCutcheon & Brown 2012; Houghton et al. 2008; Watts et al. 2003; Newburn 2012; Ogden et al. 1997a; Lavender & Chapple 2005). | ‘Several of the women had first considered home birth following the community midwives’ suggestion at antenatal clinic. This was encouraging as it showed evidence of offering choice about place of birth:  “*My midwife suggested I looked into home delivery and it’s the best thing I ever done*”.’(Andrews 2004)p.519  ‘*She* [the midwife] *kind of reassured me a bit on a few things, you know, what happens in an emergency and that kind of thing. It put my mind at rest a bit after I’d spoken to her*.’  Researcher: “What were the things that were worrying you about having your baby at home?”  Jane: “*Just basically about what would happen in an emergency and you know how often they would come across this, are they equipped to deal with a baby that needs resuscitating, which they were. Also, what would be the circumstances where I would need to be transferred to hospital, things like that.. In the end we came up with the decision that it might be even safer to have the baby at home with me having such a quick labour* (last time).’ (Houghton et al. 2008) p.7 |
